# HemoSYS: A Toolkit for Image-based Systems Biology of Tumor Hemodynamics

**DOI:** 10.1038/s41598-020-58918-3

**Published:** 2020-02-11

**Authors:** Janaka Senarathna, Ayush Prasad, Akanksha Bhargava, Stacy Gil, Nitish V. Thakor, Arvind P. Pathak

**Affiliations:** 1 Russell H. Morgan Department of Radiology and Radiological Science, Johns Hopkins University School of Medicine, Baltimore, MD 21205 USA; 2 Department of Biophysics, Johns Hopkins University, Baltimore, MD 21205 USA; 3 Department of Biomedical Engineering, Johns Hopkins University School of Medicine, Baltimore, MD 21205 USA; 4 Sidney Kimmel Comprehensive Cancer Center, Johns Hopkins University School of Medicine, Baltimore, MD 21205 USA

**Keywords:** Cancer imaging, Cancer microenvironment, Tumour angiogenesis

## Abstract

Abnormal tumor hemodynamics are a critical determinant of a tumor’s microenvironment (TME), and profoundly affect drug delivery, therapeutic efficacy and the emergence of drug and radio-resistance. Since multiple hemodynamic variables can simultaneously exhibit transient and spatiotemporally heterogeneous behavior, there is an exigent need for analysis tools that employ multiple variables to characterize the anomalous hemodynamics within the TME. To address this, we developed a new toolkit called HemoSYS for quantifying the hemodynamic landscape within angiogenic microenvironments. It employs multivariable time-series data such as *in vivo* tumor blood flow (BF), blood volume (BV) and intravascular oxygen saturation (Hb_sat_) acquired concurrently using a wide-field multicontrast optical imaging system. The HemoSYS toolkit consists of propagation, clustering, coupling, perturbation and Fourier analysis modules. We demonstrate the utility of each module for characterizing the *in vivo* hemodynamic landscape of an orthotropic breast cancer model. With HemoSYS, we successfully described: (i) the propagation dynamics of acute hypoxia; (ii) the initiation and dissolution of distinct hemodynamic niches; (iii) tumor blood flow regulation via local vasomotion; (iv) the hemodynamic response to a systemic perturbation with carbogen gas; and (v) frequency domain analysis of hemodynamic heterogeneity in the TME. HemoSYS (freely downloadable via the internet) enables vascular phenotyping from multicontrast *in vivo* optical imaging data. Its modular design also enables characterization of non-tumor hemodynamics (e.g. brain), other preclinical disease models (e.g. stroke), vascular-targeted therapeutics, and hemodynamic data from other imaging modalities (e.g. MRI).

## Introduction

The tumor microenvironment (TME) often exhibits anomalous hemodynamics that include spatiotemporally heterogeneous changes in blood flow, blood volume, oxygen saturation, and blood rheology at the microvascular scale^1,2^. In addition, the process of *de novo* microvessel formation to sustain tumor growth or angiogenesis^3^, results in blood vessels with irregular diameters, without geometric hierarchy, arteriovenous shunts, poor smooth muscle lining and hyper-permeability^4^. These blood vessels result in TME- or system-wide hemodynamic abnormalities that are inherently multi-variable in nature^5^, and are characterized by acute hypoxia^2^, heterogeneous cancer cell proliferation^6^, enhanced metastatic potential^7^, restricted drug delivery^8^, and resistance to radiotherapy^7^. Therefore, characterizing a tumor’s vascular phenotype, not just with conventional structural markers of angiogenesis such as microvessel density, vessel caliber and tortuosity^4^, but with indices of  hemodynamic heterogeneity could yield crucial new insights into its vascular systems biology.

To conduct dynamic “systems-level” characterizations, one first needs to acquire multiple hemodynamic variables (i.e. multivariable) *in vivo*, followed by spatiotemporal analyses. In contrast to MRI, PET, CT and ultrasound^1^, optical contrast mechanisms^9,10^ can be easily combined to acquire multiple hemodynamic variables *in vivo*. However, the lack of analytical tools for quantifying spatiotemporal changes in multiple hemodynamic variables has limited our ability to rigorously characterize the vascular phenotype within the *in vivo* TME. For example, optical imaging techniques such as laser speckle contrast imaging^11^ and optical coherence tomography^12^ have been used to image *in vivo* microvascular blood flow^10,13,14^; intrinsic optical signal^15^ and photoacoustic^16^ imaging have been used to image *in vivo* microvascular oxygen saturation^17,18^ and blood volume^10,19^, while various fluorescence imaging techniques have been employed to interrogate other hemodynamic variables such as the *in vivo* hematocrit, shear-stress and leukocyte-endothelial cell interactions, to name a few^20,21^. These imaging techniques generate an unprecedented volume (~GB) of multivariable data describing spatial and temporal hemodynamic changes within the TME. However, most image-analysis pipelines are ill-equipped to handle such multivariable hemodynamic data. This is because current approaches focus on single variable analyses^13,17^ instead of spatiotemporal analyses of multiple variables. Moreover, these approaches involve either spatial analyses at discrete time points (e.g. longitudinal experimental data)^13,18^, or temporal analyses at discrete spatial locations within the TME^2,22^ (please see Supplementary Table 1 for a summary of optical imaging methods for characterizing the TME). These shortcomings limit systems-level characterizations of the TME and preclude quantifying the dynamic and transient relationships that may exist between hemodynamic variables *in vivo*.

**Figure 1:**
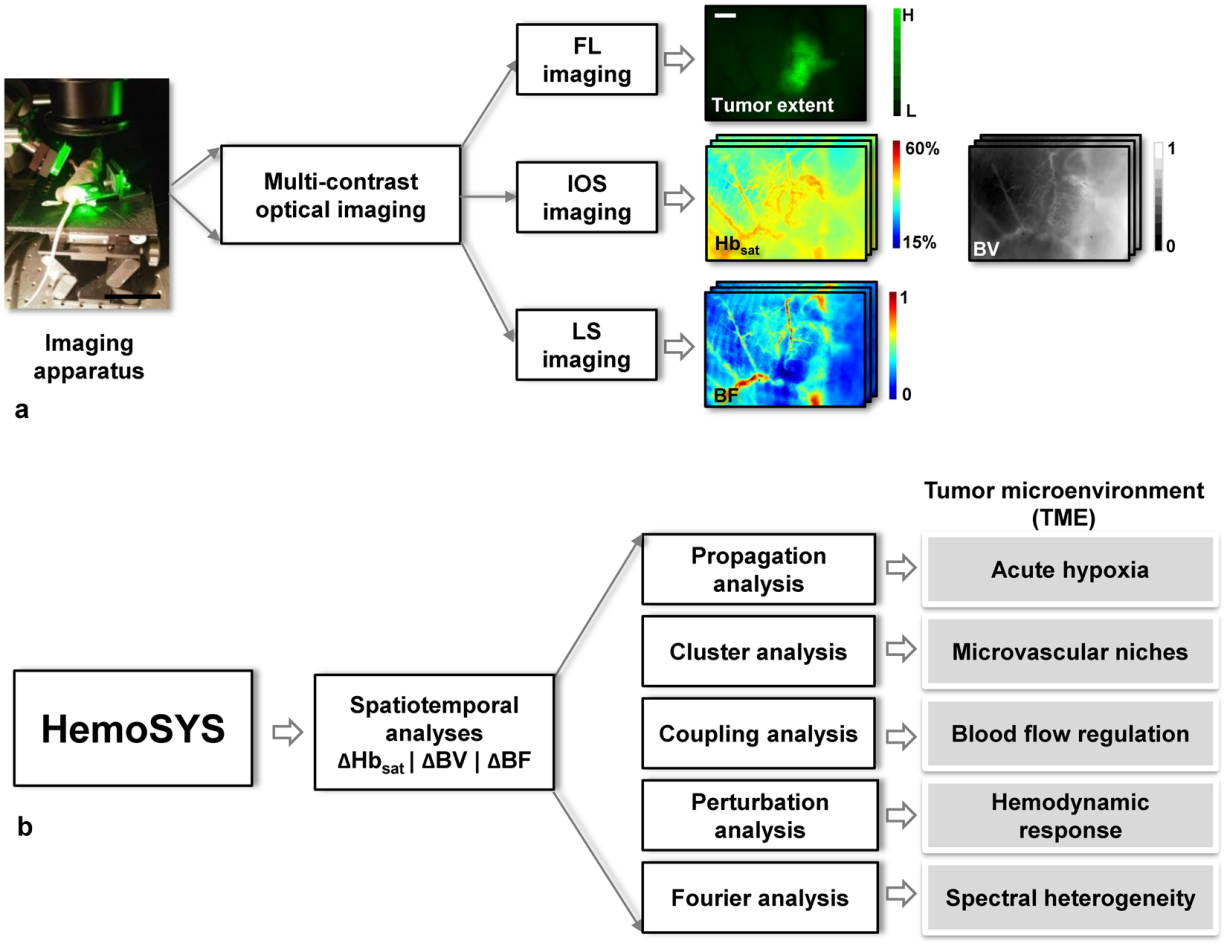
HemoSYS is a modular toolkit for systems-level characterization of tumor hemodynamics. (**a**) Schematic of the multicontrast optical imaging system designed to interrogate multiple tumor hemodynamic variables. First, tumor extent was identified using fluorescence (FL) imaging of GFP expressing tumor cells. To characterize the tumor microenvironment (TME), high-resolution (i.e. 5 μm) *in vivo* images of microvascular oxygen saturation (Hb_sat_), blood volume (BV), and blood flow (BF) were acquired using intrinsic optical signal (IOS) imaging and laser speckle (LS) imaging, respectively. Imaging was repeated every 30 seconds for up to an hour. (**b**) The HemoSYS pipeline for the analyses and visualization of spatiotemporal changes (Δ) in Hb_sat_, BV and BF. HemoSYS includes modules for: (i) visualizing and quantifying the expansion of acute hypoxic regions via a propagation analysis; (ii) partitioning the TME into distinct niches with unique vasodilation or vasoconstriction responses using a cluster analysis; (iii) quantifying blood flow regulation via vasodilation or vasoconstriction using a coupling analysis; (iv); using a perturbation analysis to quantify the multivariable hemodynamic response of the microvascular bed during carbogen inhalation; and (v) assessing hemodynamic heterogeneity in the frequency domain using Fourier analysis. Scale bars indicate 10 cm and 1 mm, respectively.

Therefore, we developed the HemoSYS toolkit for characterizing spatiotemporal changes in multivariable hemodynamic data (Fig. 1). HemoSYS treats the TME as a multi-component system and characterizes its spatiotemporal dynamics using fundamental engineering principles. It was designed using the powerful image processing platform MATLAB (Mathworks, MA) but does not require any programming expertise to operate. All user interactions with HemoSYS are via interactive graphical user interfaces (GUIs) that facilitate its ease-of-use by basic scientists and clinicians. HemoSYS consists of five processing modules: (i) propagation, (ii) clustering, (iii) coupling, (iv) perturbation and (v) Fourier analyses. We demonstrate the utility of each module by characterizing transient changes in microvascular oxygen saturation, tumor blood volume (i.e. vasodilation and vasoconstriction), and tumor blood flow, acquired *in vivo* using wide-field multicontrast optical imaging of an orthotopic breast cancer model. Specifically, we used HemoSYS to characterize hemodynamic dysfunction within the TME: (i) the spread of acute hypoxia (i.e. hypoxia occurring on the scale of tens of minutes); (ii) the initiation and dissolution of vascular niches exhibiting distinct vasodilation or vasoconstriction transients; (iii) poor blood flow regulation; (iv) non-uniform hemodynamic responses to systemic perturbations (e.g. carbogen gas inhalation); and (v) the heterogeneity of hemodynamic power spectra within the TME. HemoSYS also serves as a unifying framework for previously reported functional analysis approaches described by other investigators in^16,17,23^. The HemoSYS toolkit is available for free download via http://www.pathaklab.org/HemoSYS. Basic scientists and clinicians alike can use it as a complementary tool for analyzing time-series hemodynamic data acquired using various *in vivo* imaging methods. Users proficient in programming can also download a ‘developer’s version’ of the toolkit for easy editing, customization and integration into their own image acquisition, hardware or real-time analysis pipelines. We expect the HemoSYS toolkit to help researchers and clinicians better understand the role of microvascular hemodynamics and associated phenomena in cancer, as well as other diseases involving aberrant vasculature and hemodynamics.

## Methods

The ensuing sections describe the HemoSYS toolkit, breast cancer model, multicontrast imaging system, *in vivo* imaging protocols, and the pre-processing steps used.

### Operation of the HemoSYS toolkit

HemoSYS is designed using the MATLAB (MathWorks, MA) software platform. A description of how to use the toolkit can be found in the ‘HemoSYS User Guide’ included with the Supplementary Material. Briefly, before starting HemoSYS, the user is required to create a data folder that conforms to the HemoSYS data structure described in Supplementary Fig. 1a, wherein the image files are organized first by experiment or trial number, and second by the physiological variable being interrogated. As illustrated in Supplementary Fig. 1b, double-clicking the ‘HemoSYS.exe’ icon after downloading and installing it, initiates a GUI that prompts the user to select one of five data analysis modules. Next, the user is prompted to select the folder in which the input data resides. Experiments/trials and physiological variables will be automatically read in by HemoSYS. Representative usage of each HemoSYS module using *in vivo* BF, BV and Hb_sat_ data acquired from an orthotropic breast cancer model is described in the ensuing sections.

### Propagation analysis (HemoSYS Module 1)

**Figure 2:**
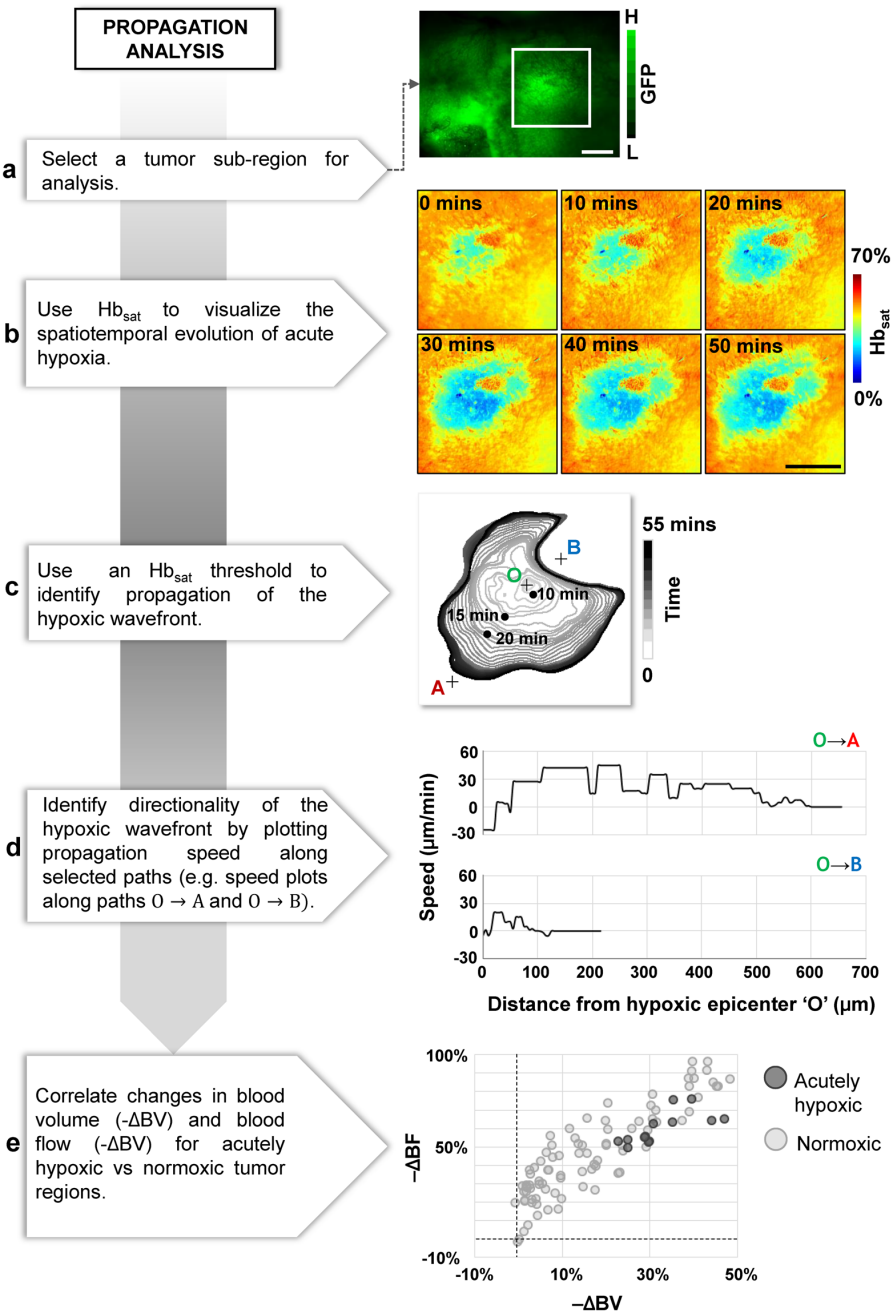
Characterizing acute hypoxia via the propagation analysis module. (**a**) A region within the tumor FoV was selected for propagation analysis. (**b**) Time lapse images of microvascular oxygen saturation (Hb_sat_) within the selected region. (**c**) A minute-by-minute depiction of the expanding wavefront of the hypoxic zone. The contours are color coded using a grayscale color map in which darker contours correspond to later times. The point ‘O’ was selected as the origin, and ‘A’, and ‘B’ selected to define two directions (O → A and O → B). (**d**) Distinct expansion-rate (i.e. speed) profiles of the hypoxic zone along O → A and O → B. (**e**) A scatter plot illustrating the reduction in blood flow (−ΔBF) versus the reduction in blood volume (−ΔBV) for sub-regions within the acutely hypoxic (dark gray circles) and normoxic (light gray circles) zones, as defined by the first (T = 0 mins) and last (T = 55 mins) contours in (**c**). Scale bar = 1 mm in all panels.

We employed Hb_sat_ as a surrogate of *in vivo* tissue oxygenation and assessed the propagation dynamics of an acute hypoxic event. Figure 2 shows a schematic of the image analysis pipeline. First, we used an Hb_sat_ signal threshold of 30% to identify the boundaries of the hypoxic wavefront at each 1 minute time step. Next, we calculated the speed of the propagating hypoxic wavefront from the distance between each successive pair of wavefronts along a preselected propagation direction. We also quantified the reduction in blood flow (i.e. −ΔBF) and any concomitant decrease in blood volume (i.e. −ΔBV) within 50 × 50 pixel sub-regions of the tumor field of view (FoV) and generated a scatter plot showing the relationship between -ΔBF vs. −ΔBV across all sub-regions.

### Cluster analysis (HemoSYS Module 2)

**Figure 3:**
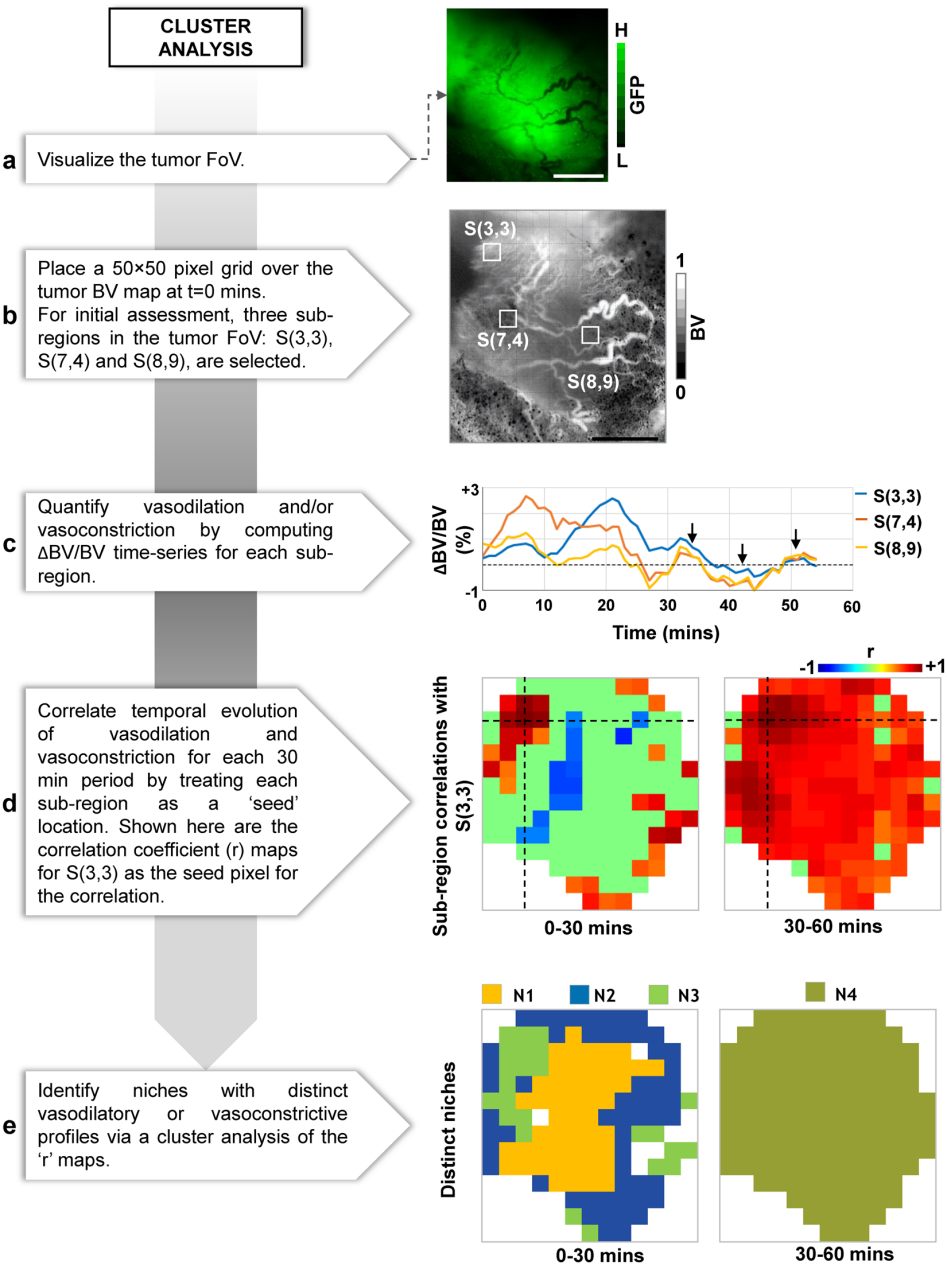
Identifying emergent microvascular niches via the cluster analysis module. (**a**) Image of the fluorescent tumor, and (**b**) the corresponding blood volume (BV) map at T = 0 mins. The grid overlaid on the BV map shows 50 × 50 pixel sub-regions (S) for which average time-series were computed for further analysis. (**c**) Time-series from three representative sub-regions: S[3, 3], S[7, 43], S[8, 9], where the top left corner of the image is the origin. Black arrowheads indicate the merging of time-series during the final 30 mins of imaging. (**d**) Maps showing the temporal correlation coefficient (r) between the ΔBV/BV time-series of a seed sub-region S[3, 3] (black cross-hairs) and each tumor sub-region during 0–30 mins and 30–60 mins of imaging. (**e**) Results of the cluster analysis identify tumor niches with unique vasodilatory and vasoconstriction temporal profiles for the 0–30 mins and 30–60 mins periods Scale bar = 1 mm in all panels.

We performed a cluster analysis using the BV time-series to identify the initiation and dissolution of niches that exhibited distinct vasodilation or vasoconstriction dynamics within the tumor FoV. Figure 3 shows a schematic of the image analysis pipeline. First, we computed the degree of vasodilation/vasoconstriction as the fractional change in blood volume (i.e. ΔBV/BV) for each 50 × 50 pixel sub-region of the tumor FoV at 1 minute time intervals. Next, we computed the spatial distribution of correlation coefficients (r) between the vasodilation and vasoconstriction time-series for a seed 50 × 50 pixel sub-region and every other 50 × 50 pixel sub-region within the tumor FoV. This step was repeated until correlation maps were created by using each sub-region in the tumor FoV as a seed location. We then linearized each of these 2D correlation maps (i.e. rearranged into 1D vectors) and stacked them to create a ‘master correlation matrix’ which showed the correlation between time-series of any two sub-regions. Following the clustering algorithm described by White *et al*.^24^, we then applied singular value decomposition (SVD) to create a set of initial clusters. The final vascular niches were identified by iteratively refining these clusters via a correlation analysis (See White *et al*.^24^ for an excellent description of the clustering algorithm). A correlation threshold of 0.7 and a minimum cluster size of 5% of the tumor FoV were used to reduce the impact of noise.

### Coupling analysis (HemoSYS Module 3)

**Figure 4:**
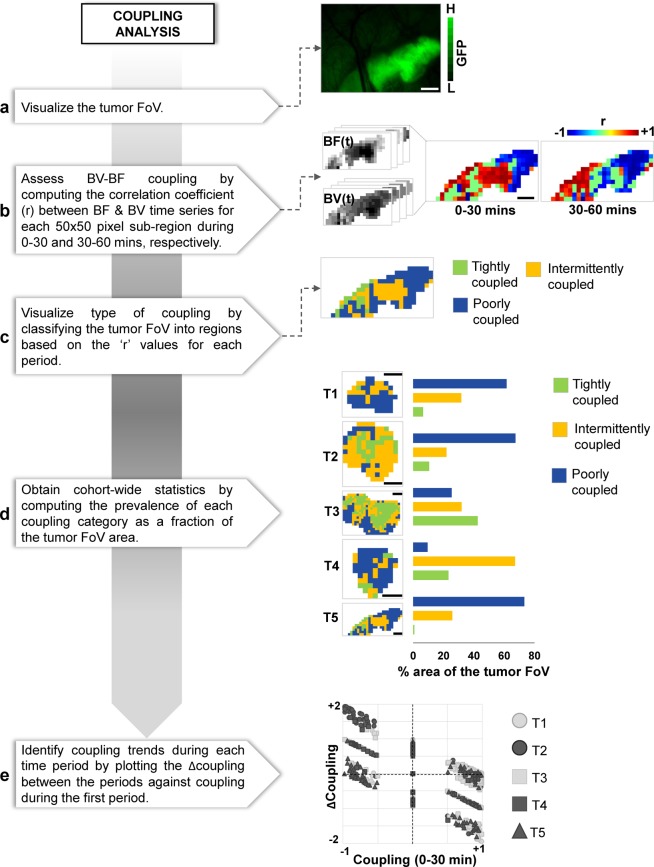
Quantifying tumor blood flow regulation via the coupling analysis module. (**a**) Image of the fluorescent tumor. (**b**) Image stacks corresponding to the tumor blood flow (BF) and blood volume (BV) time-series for the FoV indicated in (**a**). Maps of the correlation coefficient (r) between BF and BV time-series (i.e. the coupling between these two quantities) for each 50 × 50 pixel sub-region in the tumor FoV for 0–30 mins and 30–60 mins intervals. (**c**) Correlation coefficient maps were used to classify the tumor FoV into regions that were (i) tightly coupled (r > 0.7 during both time intervals, i.e. normal blood flow regulation); (ii) poorly coupled (r < 0.7 during both time intervals, i.e. uncoupled); and (iii) intermittently coupled (i.e. normal blood flow regulation during one time interval but not the other). One can select an appropriate threshold as desired. (**d**) Maps of coupling categories were generated for a cohort of 5 tumors (T1–T5). The percent tumor area occupied by each category is also indicated. (**e**) Scatter plot showing the change (Δ) in coupling from 0–30 mins duration to 30–60 mins duration versus their initial coupling (i.e. during 0–30 mins) for each 50 × 50 pixel sub-region in all 5 tumors. Scale bar = 1 mm in all panels.

We used a coupling analysis to characterize the degree of BF regulation via local vasodilation or vasoconstriction within the tumor FoV. Figure 4 shows a schematic of the image analysis pipeline. We defined the coupling between two hemodynamic variables to be the correlation coefficient (r) between their time-series. By computing ‘r’ for BF and BV time-series at each 50 × 50 pixel sub-region, we created maps illustrating the spatial distribution of BF-BV coupling within the tumor FoV. We also assessed whether this BF-BV coupling changed over time, i.e. by comparing ‘r’ between two successive 30 min intervals. We classified the tumor FoV into regions that showed a tight BF-BV coupling (i.e. r > 0.7 during both periods), regions that were poorly coupled (i.e. r < 0.7 during both periods), and regions that showed intermittent coupling (i.e. r > 0.7 in one period and r < 0.7 during the other). Moreover, to determine if there was any trend in BF-BV coupling, we plotted the change in BF-BV coupling during the two periods (i.e. r[30–60 min] − r[0–30 min]) relative to the BF-BV coupling during the 1^st^ 30 min (i.e. r[0–30 min]) for each 50 × 50 pixel sub-region in the tumor FoV.

### Perturbation analysis (HemoSYS Module 4)

**Figure 5:**
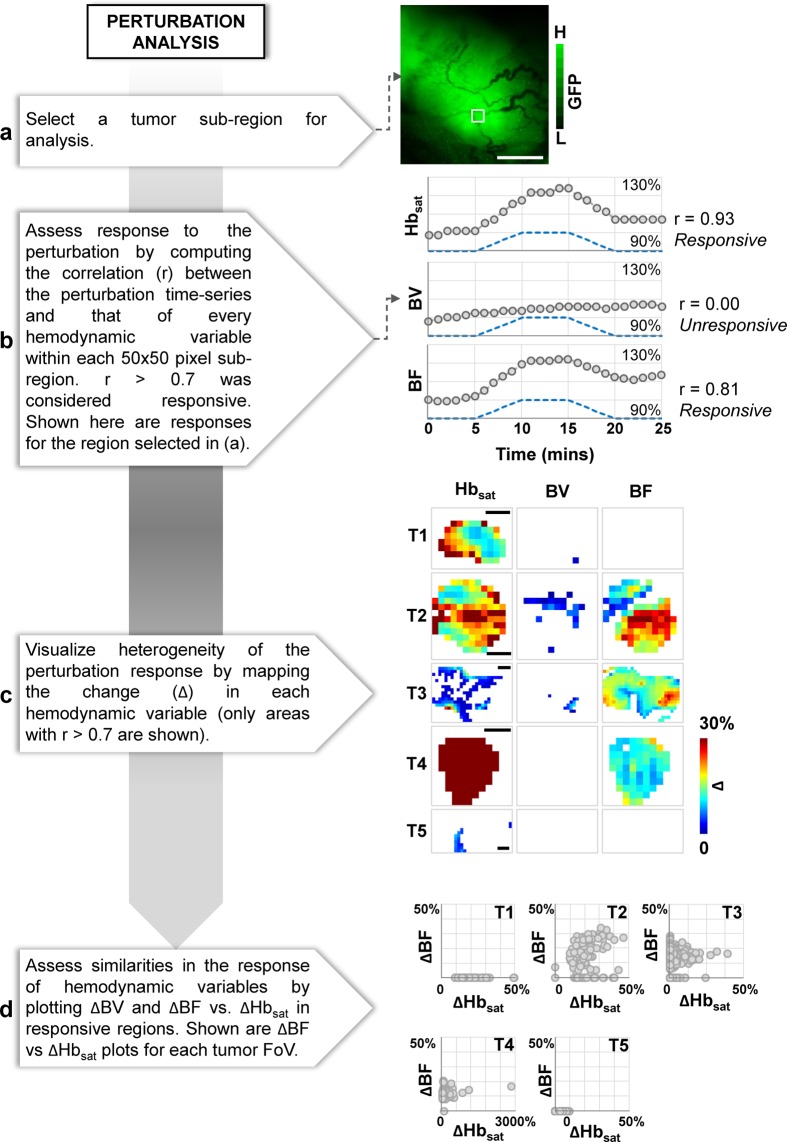
Characterizing the response of multiple hemodynamic variables to a systemic perturbation via the perturbation analysis module. (**a**) Image of the fluorescent tumor. (**b**) The multivariable hemodynamic response to a systemic perturbation with carbogen. Time-series for the perturbation (dashed line) and each hemodynamic response (grey circles) for the sub-region indicated by the white square in (**a**). Carbogen was administered from 10–20 mins. (**c**) Maps showing the response of each hemodynamic variable (i.e. ΔHb_sat_, ΔBF and ΔBV) to carbogen for a cohort of 5 tumors (T1–T5). (**d**) Scatter plots of ΔBF vs. ΔHb_sat_ for each responsive sub-region within each tumor. Scale bar = 1 mm in all panels.

We mapped the spatial distribution of the response of multiple hemodynamic variables to a systemic perturbation (e.g. carbogen inhalation) via a two-step process. Figure 5 shows a schematic of this image analysis pipeline. First, we computed the correlation coefficient (r) between the perturbation (i.e. reference) time-series and that of each hemodynamic variable within 50 × 50 pixel sub-regions of the tumor FoV. The perturbation time-series in this case was generated by filtering the carbogen inhalation paradigm using pre-processing steps that were identical to that for the hemodynamic time-series. We then used a threshold of r > 0.7 to identify strongly correlated or ‘responsive’ tumor sub-regions. Next, we computed the magnitude (Δ) of the response for each sub-region. Here, Δ was the difference between the mean hemodynamic levels during baseline and the perturbation period. We also generated scatter plots comparing the response levels between hemodynamic variables in different tumor sub-regions.

### Fourier analysis (HemoSYS Module 5)

**Figure 6:**
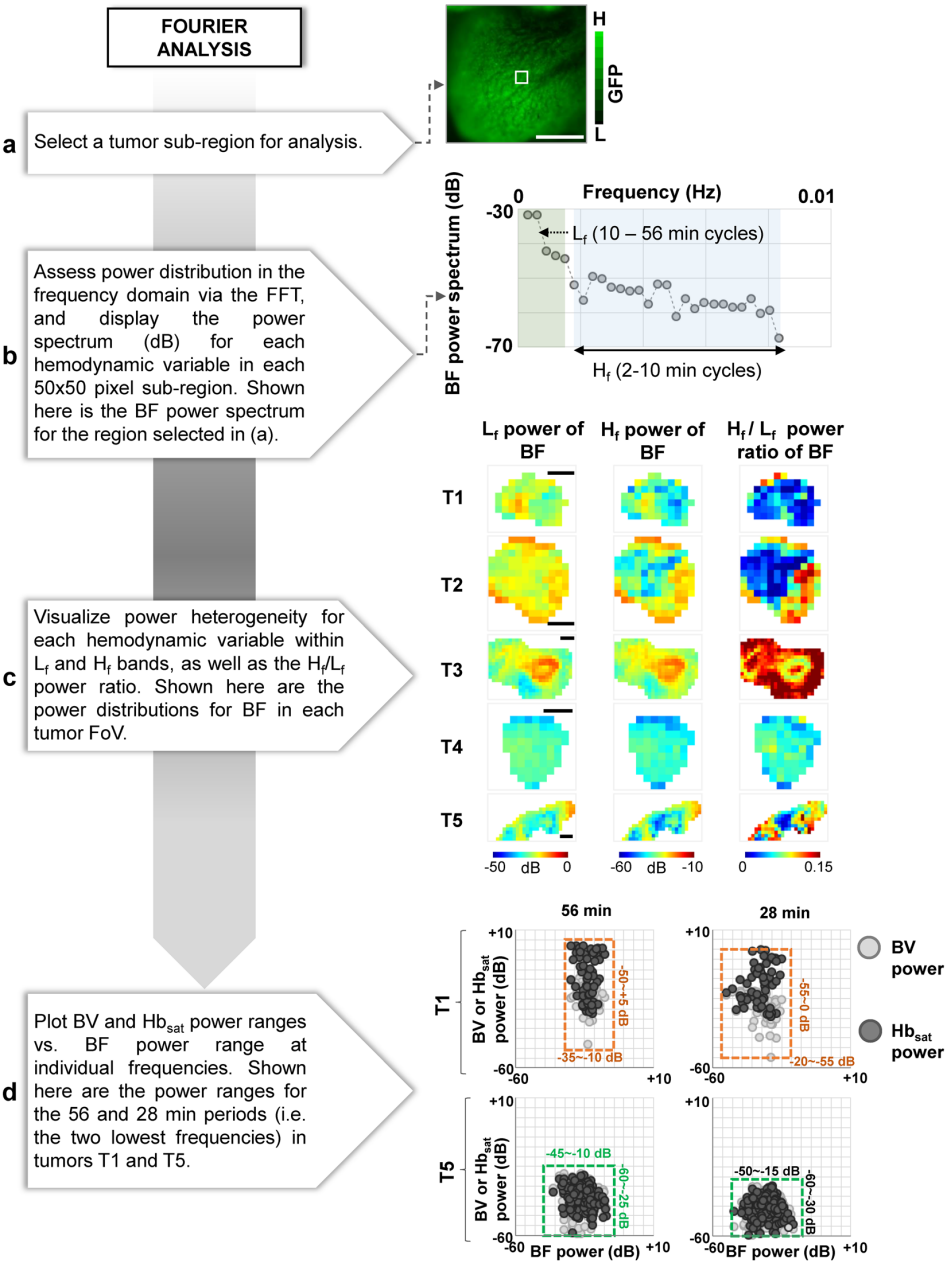
Characterizing the heterogeneity of tumor hemodynamics in the frequency domain via the Fourier analysis module. (**a**) Image of the fluorescent tumor. (**b**) A schematic illustrating the power spectrum of blood flow (BF) transients from the selected sub-region indicated by the white box in (**a**). A low frequency band (L_f_, light green) corresponding to the 10–56 min period and a high frequency band (H_f_, light blue) corresponding to a 2–10 min period were identified. (**c**) A panel showing maps of: power of BF transients in the L_f_ band, power of BF transients in the H_f_ band, and the ratio between powers of BF transients in H_f_ and L_f_ bands (i.e. H_f_/L_f_) for each 50 × 50 pixel sub-region in five tumors T1-T5. (**d**) Scatter plots showing the power of BV (light gray) and Hb_sat_ (dark gray) transients vs. the power of BF transients at each sub-region for the 56 and 28 minute period for tumors T1 and T5. Hashed boxes with horizontal and vertical labels indicate the range of BF, BV and Hb_sat_ powers. Green boxes indicate BV and Hb_sat_ ranges less than or similar to BF, while orange boxes indicate the opposite. Scale bar = 1 mm in all panels.

We performed a Fourier analysis to characterize transient changes in the power spectra of hemodynamic variables. Specifically, we used the Fast Fourier Transform (FFT) and computed the power spectra for each hemodynamic variable in a 60 minute period over 50 × 50 pixel sub-regions of the tumor FoV. We used a 56-point FFT due to the use of a 5 minute mean filter with a 1 minute step size during pre-processing. Transients with periods ≤ 1 minute were not analyzed because they were filtered out during the pre-processing stage. Figure 6 shows a schematic of this image analysis pipeline. For simplicity, we examined a low frequency band (L_f_) that corresponded to periods of 10–56 minutes, and a high frequency band (H_f_) that corresponded to periods of 2–10 minutes. We first mapped the power in the L_f_ and H_f_ bands for all tumor FoVs. These were computed as a dB value relative to the mean level for each hemodynamic variable. We also computed the ratio of the power in the high and low frequency bands (i.e. power in H_f_/power in L_f_). Moreover, for certain individual frequencies, we used 2D scatter plots to compare the relationship among Hb_sat_, BV and BF power. We annotated the scatter plots with hashed boxes and vertical/horizontal labels to indicate power ranges. The green box indicates BV and Hb_sat_ ranges less than or similar to BF, while the orange box indicates the opposite. Power values ≤ −60 dB were treated as noise and omitted.

### Animal preparation

All animal experiments were conducted in accordance with an approved Johns Hopkins University Animal Care and Use Committee (JHU ACUC) protocol. The Johns Hopkins University animal facility is accredited by the American Association for the Accreditation of Laboratory Animal Care, and meets the National Institutes of Health standards as set forth in the “Guide for the Care and Use of Laboratory Animals”. We inoculated five female athymic nude mice (~20 g) with 200 K MDA-MB-231 cells engineered to stably express green fluorescence protein (GFP) in the lower mammary fat pad. Tumors were monitored until they reached ~2–3 mm in diameter. Each mouse was then anesthetized with an intraperitoneal injection of a mixture of ketamine (90 mg/kg), xylazine (5 mg/kg) in saline. Next, the tumor was surgically exposed via a skin flap and a 3D-printed, 10 mm diameter window attached for *in vivo* multicontrast optical imaging. Finally, the mouse was transferred to a custom-designed imaging platform and maintained under isoflurane anesthesia (2% isoflurane at 0.5–1 L/min) for the duration of the experiment.

### Multicontrast optical imaging system

We used a custom built multicontrast optical imaging platform to interrogate the TME of an orthotopic breast cancer model. Supplementary Fig. 2 shows a schematic of this system. Three image contrast mechanisms: fluorescence (FL)^25^, hemoglobin absorption-based intrinsic optical signal (IOS)^15^ and laser speckle (LS)^11^ were used. The surgically implanted window provided stable optical access to the breast cancer xenograft during *in vivo* imaging. The tumor extent was identified using FL imaging of GFP expressing breast cancer cells. The tumor’s microvascular oxygen saturation (Hb_sat_) and blood volume (BV, indicative of microvascular architecture) were imaged with IOS, while tumor blood flow (BF) was imaged with LS. For a description of the imaging system, please see Supplementary Methods.

### *In vivo* image acquisition

We first acquired FL images to identify the tumor extent. Next, we acquired images with IOS (10 images at 100 ms exposure time under 570 nm and 600 nm illumination, respectively) and LS (40 images at 100 ms exposure time) at approximately 30 s intervals for an hour during inhalation of room air (mixed with 2% isoflurane). Images were acquired at 5 μm spatial resolution over a 5 × 7 mm^2^ FoV. We used the same *in vivo* imaging protocol during the carbogen gas (95% O_2_ and 5% CO_2,_ with 2% isoflurane at 1 L/min) inhalation paradigm (10 minutes each of room air, carbogen and room air inhalation, respectively). The body temperature of the mice was maintained during imaging using a heating pad.

### Pre-processing steps

Pre-processing steps for generating spatial maps of Hb_sat_, BV and BF at each imaging time point are described in Supplementary Methods. Supplementary Fig. 3 shows a schematic of the pre-processing pipeline. Next, an FoV was chosen for each tumor xenograft (see Supplementary Methods and Supplementary Fig. 4), and the hemodynamic time-series at each pixel filtered with a continuous mean filter in the time domain (1 minute step size, 5 minute long kernel) to reduce noise before being input to the HemoSYS modules. Most modules also employed a discrete mean filter in the spatial domain (50 × 50 pixels) to provide resilience against microscopic motion that can occur when imaging a soft tissue bed such as the mammary fat pad.

## Results

### HemoSYS is a modular toolkit for systems-level characterization of tumor hemodynamics

Using a custom-built multicontrast optical imaging platform, we imaged the *in vivo* hemodynamics within the TME of an orthotopic breast tumor model at 5 μm spatial resolution over a 5 × 7 mm^2^ FoV. We identified tumor extent via FL imaging of GFP expressing tumor cells, and computed *in vivo* maps of microvascular oxygen saturation (Hb_sat_) and blood volume (BV) via IOS imaging, and blood flow (BF) via LS imaging (Fig. 1a). We also acquired the same hemodynamic signals during a 30 min carbogen gas inhalation protocol. This multicontrast, wide-area imaging data enabled us to quantify the spatiotemporal evolution of *in vivo* tumor hemodynamics. We then applied five distinct system analysis modules in the HemoSYS toolkit to quantify and visualize changes in these hemodynamic variables (Fig. 1b).

First, we imaged an acutely hypoxic TME region and used propagation analysis to quantify its expansion. Second, we used cluster analysis to identify unique microvascular niches within the tumor FoV that exhibited distinct vasodilation or vasoconstriction dynamics. Third, we quantified the extent and consistency of blood flow regulation by local vasodilation or vasoconstriction using a coupling analysis. Fourth, we used a perturbation analysis to identify hemodynamic response patterns to carbogen gas inhalation. Finally, we went beyond spatiotemporal analyses and used Fourier analysis to characterize changes in the power spectra of each hemodynamic variable.

### Propagation analysis - characterizing the spatiotemporal evolution of acute hypoxia

Propagation analysis of a hypoxic event using HemoSYS enabled us to characterize its temporal and directional dynamics. Figure 2a shows a representative FL image wherein the tumor extent was identified via GFP expression of breast cancer cells. A region within the tumor was selected for analysis (Fig. 2a, white box). Time-lapse images illustrate the spatial evolution of Hb_sat_ levels within this region over an hour, and enabled us to observe expansion of the hypoxic region *in vivo* (Fig. 2b). The contours of the hypoxic wavefront permitted identification of the direction of hypoxia propagation as shown for the cropped region in Fig. 2c. This contour map also enabled us to identify paths of least (O → A) and maximal (O → B) resistance to hypoxia propagation. As shown in Fig. 2d, we observed unique, path-specific, temporal dynamics of hypoxia propagation. Finally, a comparison between the degree of blood flow (−ΔBF) and blood volume (−ΔBV) attenuation for hypoxic and normoxic regions (Fig. 2e) revealed that regions within this tumor exhibiting acute decreases in Hb_sat_ (i.e. acutely hypoxic regions) also exhibited reductions in BF and BV. Collectively, these data implied that the spreading hypoxic wave within this tumor region was largely due to the lack of perfusion.

### Cluster analysis - identifying emergent microvascular niches within the TME

A cluster analysis using HemoSYS enabled identification of the emergence and dissolution of microvascular niches with unique vasodilatory or vasoconstriction dynamics. Figure 3a,b show a FL image of the tumor and its microvascular topology. The ΔBV/BV time-series during the first 30 minutes for different sub-regions indicate the existence of spatially and temporally unique vasodilatory or vasoconstrictive profiles within the same tumor (Fig. 3c). However, the differences between these profiles diminished during the next 30 minutes of imaging (Fig. 3c, arrows). We generated maps (Fig. 3d) of the correlations (r) between the ΔBV/BV time-series at a ‘seed’ pixel (black cross-hairs) and every other sub-region within the tumor FoV computed for each 30 minute period. We observed that a small fraction of the tumor exhibited positive correlation coefficients during the first 30 minutes, which increased to a substantial fraction during the next 30 minutes. By employing a clustering algorithm, we observed that this tumor exhibited three distinct microvascular niches (N1, N2, N3) during the first 30 minutes of imaging, which eventually merged into a single niche (N4) during the final 30 minutes (Fig. 3e).

### Coupling analysis - quantifying the *in vivo* regulation of tumor blood flow

Computing the coupling between BF and BV dynamics in sub-regions within the tumor using HemoSYS enabled us to quantify the degree to which tumor BF was regulated via local BV changes. As shown in Fig. 4a,b, BF-BV coupling was spatially and temporally heterogeneous. Classifying tumor regions based on the spatiotemporal patterns of BF-BV coupling allowed us to identify: (i) tumor areas that exhibited tight local BF regulation, (ii) areas that showed poor local BF regulation, and (iii) areas in which BF was intermittently regulated (Fig. 4c). We observed that our cohort of 5 tumors mostly exhibited either poorly or intermittently regulated BF (Fig. 4d). Moreover, for each tumor xenograft, we observed an inverse relationship between the degree of BF regulation during the first 30 minutes, and the change in this regulation between the first and second 30 minute period (Fig. 4e).

### Perturbation analysis - characterizing the response of multiple hemodynamic variables to a systemic perturbation

Perturbation analysis using HemoSYS enabled us to quantify how tumor Hb_sat_, BV and BF uniquely responded to carbogen inhalation. Figure 5b illustrates an example of the multivariable hemodynamic response of a tumor sub-region (Fig. 5a, white box). Here, tumor Hb_sat_ and BF changed in response to carbogen inhalation while BV did not. We observed that responsive tumor areas differed between hemodynamic variables (Fig. 5c). As shown in Fig. 5c, even for a single hemodynamic variable (e.g. Hb_sat_) in each tumor xenograft (e.g. T1), the response was heterogeneous. We also observed differences in the magnitude and spatial extent in the response between tumor xenografts (Fig. 5c). Furthermore, as shown in Fig. 5d the BF response (i.e. ΔBF) and the ΔHb_sat_ responses were either uncorrelated or poorly correlated, implying that these hemodynamic variables were likely uncoupled in these areas.

### Fourier analysis – characterizing heterogeneity of tumor hemodynamics in the frequency domain

Fourier analysis using HemoSYS enabled characterization of the frequency spectrum of each hemodynamic transient within the tumor. Figure 6a,b illustrates the frequency-dependent power of a BF time-series. Using spatial maps of power in different frequency bands, we observed that power in L_f_ and H_f_ bands was spatially heterogeneous within each tumor. Figure 6c illustrates this for BF time-series. Moreover, tumor #3 (i.e. T3) exhibited the largest BF power in the L_f_ band. T3 was also the only xenograft in which we observed acute hypoxia (i.e. Fig. 2a,b). In addition, areas in T3 with the largest L_f_ band BF power corresponded to areas that exhibited acute hypoxia (Supplementary Fig. 5). BF power in the H_f_ band was also elevated in T3. In addition, Fig. 6c also illustrates the inter-tumoral heterogeneity (i.e. among tumor FoVs T1 to T5) in hemodynamic power spectra. Moreover, as shown in Fig. 6d for tumors T1 and T5, Hb_sat_ (light gray), BV (dark gray) and BF power for transients with 28 and 56 minute periods exhibited distinct relationships with each other. For example, for both periods in tumor T1, BF power spanned a smaller range relative to the Hb_sat_ and BV power (orange hashed box), while in tumor T5 BF power spanned a larger or similar range to that of the Hb_sat_ and BV power (green hashed box).

## Discussion

We developed a new modular toolkit called HemoSYS for image-based systems analysis of *in vivo* hemodynamics. HemoSYS enables the characterization of spatiotemporally heterogeneous hemodynamic variables via five processing modules. HemoSYS is an easy-to-use toolkit for basic scientists and clinicians because the user interacts with it via a GUI and does not require any programming expertise to operate it. Here we described its implementation and demonstrated its usefulness in quantifying hemodynamic abnormalities within the TME of a preclinical breast cancer model.

### Propagation analysis (HemoSYS Module 1)

We used the propagation analysis module to characterize the *in vivo* expansion of an acutely hypoxic tumor region. Previous reports of time-series analyses of oxygenation fluctuations were conducted at preselected locations within the TME^2,17,18,26^. HemoSYS significantly enhances this analysis by offering: (i) wide-field time lapse visualizations that cover all visible tumor regions; (ii) contour plots to identify hypoxic wavefront propagation within the TME; and (iii) the ability to assess expansion of hypoxic areas as a function of the location and direction. Tumor biologists could use this module to characterize how acute hypoxia varies with type of cancer, tumor grade, or in response to therapy. One could also complement this module with histopathological data to determine whether genetic or molecular heterogeneity of the underlying TME correlates with the observed *in vivo* phenotype. Moreover, the propagation analysis module can be useful to researchers or clinicians interested in quantifying disruptions in physiological variables that occur in a wave-like manner, such those that occur during focal cerebral ischemia^27^ or spreading depolarizations^28^.

### Cluster analysis (HemoSYS Module 2)

The cluster analysis module provides: (i) the option to apply simple mathematical transformations to the hemodynamic time series; (ii) visualization of maps of temporal correlation coefficients for selected seed locations; and (iii) the ability to identify clusters or regions with similar hemodynamic properties. While we implemented a single variable transformation (e.g. the temporal derivative), one could modify HemoSYS (using the developer’s version) to include multivariable transformations such as the estimation of erythrocyte speed from the BF/BV ratio as well as vascular shear stress from the radial gradient of erythrocyte speeds within individual microvessels^21^. The correlation coefficient analysis implemented here was derived from that used in the resting-state fMRI literature wherein hemodynamics act as a surrogate for resting-state neuronal activity^29,30^. Here, we employed a similar paradigm to directly identify correlations between hemodynamic time-series within the TME, albeit without the presence of a neuronal substrate. Instead of conventional single channel, single time-point approaches that may be insufficient for characterizing the hemodynamic heterogeneity within the TME, scientists and clinicians could use this correlation technique to map *in vivo* alterations in local hemodynamics induced by tumor progression, therapeutics or other interventions. One could also utilize the cluster analysis to distinguish dynamic groupings of other variables such as oxygenation, blood flow, vascular permeability and tumor metabolism. Further analysis is possible by comparing dynamic clusters of two or more variables. For example, since tumor metabolism is considered a major driver of the abnormal structural adaptations occurring in tumor microvessels^31^, reconciling clusters of tissue metabolism^26^ with those of microvascular adaptation may help quantify their dynamic interplay. Moreover, comparing the dynamic clusters generated with HemoSYS to clusters created using static data (e.g. microvascular density, baseline metabolism levels etc.) could reveal additional insights into the *in vivo* TME. Additionally, users can enhance this module by incorporating faster implementations of the SVD algorithm^32,33^, alternative clustering algorithms^23^, multivariate dimensionality reduction approaches (e.g. PCA^34^, ICA^35^), as well as machine learning techniques^36,37^.

### Coupling analysis (HemoSYS Module 3)

We used the coupling analysis module to map the spatial distribution of *in vivo* BF regulation within the TME. It is well-known that different populations of tumor vessels can exhibit differential diameter changes. For example, newly formed angiogenic vessels that lack smooth muscle coverage would be less capable of regulating blood flow via diameter changes than mature vessels that may be a part of the host vasculature^5^. Our toolbox allowed us to disentangle these hemodynamic characteristics *in vivo*. Researchers can use this module to identify regional irregularities in BF regulation by examining the correlation between BF and BV. Via this approach one could classify the TME into regions that were permanently or intermittently coupled, or uncoupled. Such an analysis would be indispensable for ascertaining whether successful vascular ‘normalization’ has occurred following antiangiogenic therapy^38^. Analysis of the degree of coupling between other hemodynamic or non-hemodynamic variables could yield valuable insights into dysregulation of the TME. For example, in brain tumors, a coupling analysis between an indicator of neural activity such as fluorescence from a voltage sensitive dye^39^ or a calcium indicator expressed in the neurons^40^ and indicators of vascular function such as BV, BF or Hb_sat_, could reveal disruptions of the neurovascular unit^41,42^. Such an indicator could then be exploited as a biomarker of tumor grade, invasion, or functional restoration following therapy.

### Perturbation analysis (HemoSYS Module 4)

The perturbation analysis module enables assessing a tumor’s hemodynamic response to a systemic modulation. Here, we used carbogen gas as a systemic modulator because it is a well-characterized paradigm for studying tumor vascular biology^43^. In contrast to preclinical studies in which the response of a single hemodynamic variable is typically interrogated via imaging^43,44^, our multivariable imaging approach enables a more comprehensive characterization of the tumor’s vascular phenotype. Furthermore, the emergence of technologies such as near infrared spectroscopy^45^ and photoacoustic imaging^46^ that produce image sequences of BV (i.e. total hemoglobin concentration) and Hb_s__at_ data in cancer patients offers the exciting opportunity to use HemoSYS for assessing vascular functionality in the clinical setting.

### Fourier analysis (HemoSYS Module 5)

Finally, the Fourier analysis module permits assessing changes in the power of multiple hemodynamic variables within the TME. Tumor BF variations with cycle times longer than 1 min are generally hypothesized to underlie acute hypoxia^2,47^. In contrast to previous studies that assessed the spectral content at discrete spatial locations^17,47^, here we used the Fourier analysis module to visualize the spatial heterogeneity of slow varying BF transients and compare the magnitudes of the fluctuations among hemodynamic variables. This approach of spatially mapping the hemodynamic power spectrum would allow scientists to assess inter- and intra-tumoral heterogeneity in a manner not possible with conventional spatiotemporal analyses or single probe measurements at discrete locations within the TME. Moreover, the comparison of the power spectra of hemodynamic variables provides a simple tool for researchers to characterize inter-variable correlations in each tumor. Such tools could help cancer researchers develop new biomarkers for detecting acute hypoxia and ascertain the tumor’s propensity for such conditions. Since mature microvessels with smooth muscle are known to exhibit vascular tonicity or rhythmicity with periods of the order of tens of seconds^48^, mapping the power in BF or BV transients in an appropriate high frequency band might enable interrogation of vessel maturity within solid tumors. Additionally, higher order spectral features such as the H_f_/L_f_ power ratio could also serve as a useful input for machine learning algorithms that could be used to assess tumor grade or treatment outcomes^49^. One could also characterize the TME by mapping other spectral features such as the peak power frequency or 99% cumulative power frequency^16^, or the noise characteristics of the hemodynamic power spectra^50^. Our application of a mean filter during pre-processing ensured that high frequency components of each hemodynamic time-series were attenuated prior to their use in the HemoSYS module. In contrast, one could envision the use of hemodynamic time-series that were non-attenuated or filtered differently depending on experimental need.

### Additional considerations

All the above analyses were based on first defining tumor extent and then acquiring *in vivo* Hb_sat_, BV, and BF time-series data by combining FL, IOS, and LS imaging. These techniques do not require the administration of any exogenous contrast agents or dyes, permit imaging a large area, and were relatively easy to implement. However, by incorporating complementary imaging techniques, one could broaden the use of the HemoSYS toolkit to interrogate other aspects of the TME such as cellular metabolism^26^, tissue oxygen tension^51^, and luminal vascular volume^52^.

Moreover, the HemoSYS toolkit could be applied to preclinical hemodynamic data acquired from longitudinal (e.g. over days or weeks) *in vivo* experiments^13,26^. This would enable the assessment of hemodynamic changes with tumor progression or metastasis. A similar imaging paradigm could be implemented for patients undergoing regular imaging protocols that include the acquisition of hemodynamic time-series data (e.g. MRI, PET, and CT). With such an imaging paradigm, HemoSYS could potentially be used to quantify the effects of therapies on tumor hemodynamics, and could help design better therapeutics and identify biomarkers of therapeutic efficacy. Finally, HemoSYS could be readily modified to operate with 3D datasets from other imaging techniques such as photoacoustic^53^, magnetic resonance^54^ and positron emission tomography^55^ imaging, permitting its use by a broader audience.

## Conclusions

In conclusion, we believe that our modular HemoSYS toolkit for image-based systems biology of *in vivo* hemodynamics is an easy-to-use processing suite that will help researchers better characterize microvascular hemodynamics in a range of preclinical models. Concurrently characterizing the spatiotemporal fluctuations of a wide array of hemodynamic variables within the TME has the potential to revolutionize our understanding of the underlying pathophysiology of cancer. Such tools could prove indispensable when testing new therapeutic strategies or developing novel biomarkers of the TME. Moreover, the modular design of HemoSYS makes it generalizable for analyzing similar data acquired from other imaging (e.g. MRI, PET) modalities, as well as those measured from non-tumor tissues. Here we laid the groundwork and provided the motivation for adopting spatiotemporal analyses of multiple tumor hemodynamic variables. We hope this approach inspires the broader scientific community to develop more sophisticated analysis modules and apply them to gain new insights into a range of disease models in which the microcirculation plays a critical role.

## Supplementary information

Supplementary Information.

Supplementary Information:HemoSYS User Guide

## Data Availability

All data supporting the findings of this study are available within the article and its supplementary information files. The Matlab code used in the manuscript will be shared upon reasonable request from the corresponding author.
