# Supplementary material for: HemoSYS: A Toolkit for Image-based Systems Biology of Tumor Hemodynamics
Source: Sci Rep. 2020 Feb 11;10:2372. doi: 10.1038/s41598-020-58918-3 (PMC7012876; doi:10.1038/s41598-020-58918-3)
Supplement: Supplementary file 1 — Supplementary Information. [file 41598_2020_58918_MOESM1_ESM.pdf]

HemoSYS: A Toolkit for Image-based Systems Biology of Tumor Hemodynamics

Janaka Senarathna[1], Ayush Prasad[2], Akanksha Bhargava[1], Stacy Gil[1], Nitish V. Thakor[3] and Arvind P. Pathak[1, 3, 4]*

[1]Russell H. Morgan Department of Radiology and Radiological Science, [2]Department of Biophysics, and [3]Department of Biomedical Engineering, [4]Sidney Kimmel Comprehensive Cancer Center, Johns Hopkins University School of Medicine, Baltimore MD 21205

Supplementary Methods

Multicontrast optical imaging system

The imaging system employed three illumination sources: FL excitation of GFP was achieved via a 473 nm blue laser (100 mW, Cobolt AB, Sweden); a white light source (NI-150, Nikon Instruments Inc., NY) coupled with two bandpass filters (570±5 nm and 600±5 nm, Thorlabs Inc., NJ) provided illumination for imaging hemoglobin absorption based IOS, while a 632.8 nm He-Ne laser (0.8 mW, Thorlabs, NJ) was used for LS imaging. A customized MATLAB program (MathWorks Inc., MA) running on a PC controlled the filter wheel (FW102C, Thorlabs, NJ) for the white light source and switching of the 632.8 nm laser via a microcontroller (Arduino Uno, Arduino.cc) and solenoid. Since FL imaging was conducted at the beginning of each imaging session, the 473 nm laser was switched ON/OFF manually. A lens set (AF Micro Nikkor 60mm 1:2.8D, Nikon Instruments Inc., NY) was used for image formation on a CCD image sensor (Infinity 3, Lumenera, ON, Canada). A 496 nm long-pass filter (FF01-496/LP-25, Semrock Inc., NY) prevented the blue excitation light from entering the imaging light path while permitting other wavelengths to pass. Finally, a custom designed (SolidWorks, Dassault Systèmes, MA), 3D printed (Dimension BST1200ES, Stratasys, MN) and surgically implanted window provided stable optical access to the breast cancer xenograft.

Computation of hemodynamic variables (microvascular oxygen saturation, blood volume and blood flow)

For the computation of hemodynamic quantities based on IOS images, we first calculated a median intensity image from the 10 images acquired under 570 nm and 600 nm illumination, respectively, at each imaging time point. We repeated this for each image acquisition. We linearly resampled the median intensity image stacks for each wavelength with a fixed 30 second time resolution using a MATLAB script (Mathworks, MA). Next, using the ImageJ[1] MultiStackReg

plugin[2], we co-registered images in the time-series to the first image for each wavelength. Then, we used the 570 nm and 600 nm IOS images in a formulation of the modified Beer-Lambert law to compute the microvascular oxygen saturation (Hb_{sat}) and total hemoglobin concentration (HbT)[3]. Briefly, concentrations of oxy-hemoglobin (HbO) and deoxy-hemoglobin (Hb) were calculated by solving the following system of equations:

$$\begin{bmatrix}[\mathrm{I}(570\ \mathrm{nm})]\\ [\mathrm{I}(600\ \mathrm{nm})]\end{bmatrix} = \begin{bmatrix}1 & \varepsilon_{\mathrm{HbO}}(570\ \mathrm{nm}) & \varepsilon_{\mathrm{Hb}}(570\ \mathrm{nm})\\ \mathrm{L} & \mathrm{L} * \varepsilon_{\mathrm{HbO}}(600\ \mathrm{nm}) & \mathrm{L} * \varepsilon_{\mathrm{Hb}}(600\ \mathrm{nm})\end{bmatrix} \times \begin{bmatrix}\mathrm{HbO}\\ \mathrm{Hb}\end{bmatrix} \tag{1}$$

Here, I(λ) is the natural logarithm of the ratio between the reflected light level at each pixel and a reference light level for each wavelength λ (i.e. 570 nm or 600 nm). The reference light level was calculated by manually identifying a 20x20 pixel region from an area devoid of visible blood vessels in each tumor xenograft, and multiplying the reflected light level from this region by a nominal factor of 2. This reference light level was an estimate of the light level incident on the tumor xenograft. Incorporation of this estimate enabled robust calculations of Hb and HbO irrespective of fluctuations in the incident light levels. The pair of ε values for each wavelength λ denote the absorption coefficients of Hb and HbO. These were calculated by using the tabulated molar extinction coefficients found in [4], averaging the extinction coefficients over a ± 4 nm bandwidth around the two wavelengths (to account for finite filter bandwidth), and multiplying by a factor of 2.303 to convert extinction coefficients into absorption coefficients [4]. L(λ) is the differential path length at each wavelength λ. For simplicity, L(570 nm) was assumed to be unity. A nominal value of 4 was used for L(600 nm).

Both the multiplication factor for incident light level calculation and L(600 nm) were estimated using a Monte Carlo simulation[5] of photon migration in a simple air-tissue model. Optical properties of the tissue were modeled using hemoglobin extinction coefficients as described above, an Hb_{sat} level of 50% to indicate poor oxygenation status in tumor tissue, and an estimated anisotropy of 0.9 [6]. A scattering coefficient of 10/mm was used. We first assumed the tissue to

contain no blood vessels (i.e. a zero HbT level) to model tissue void of blood vessels. Then, using the Monte Carlo simulation, we computed the ratio between the amount of photons entering the tissue after specular reflection and the amount of photons diffusely reflected back from the tissue. We obtained a value of 2.3 which was approximated to 2 to account for uncertainties in modeling parameters. We used this as the multiplication factor to convert the imaged intensity of light from a selected tissue area void of visible blood vessels into an estimate of the incident light level. Next, we used an HbT level of 80 μM [6] to model tissue with blood vessels. Since we already denoted L(570 nm) as unity, L(600 nm) was estimated as the ratio of differential path lengths computed using the Monte Carlo simulation for 600 nm and 570 nm photons, respectively. The computed ratio of 3.6 was approximated to 4 to account of uncertainties in modeling parameters. One could perform more detailed Monte Carlo simulations of photon migration in tissue to potentially arrive at more accurate estimates.

Next, we computed Hb_{sat} and HbT according to:

$$\mathrm{Hb_{sat}} = \frac{\mathrm{HbO}}{\mathrm{HbT}}, \quad \mathrm{HbT} = \mathrm{HbO} + \mathrm{Hb} \tag{2}$$

Assuming constant hematocrit, HbT served as a surrogate of blood volume (BV). We also computed images of relative blood flow (BF) from LS images by quantifying the speckle contrast (k) for each pixel. Briefly, speckle contrast is given by[7]:

$$\mathrm{k(x,y)} = \frac{\sigma(\mathrm{x,y})}{\mu(\mathrm{x,y})} \tag{3}$$

Here, μ and σ denote the mean and standard deviation of pixel intensities across the stack of images (40 images, acquired every 30s under 632.8 nm illumination) at each pixel location (x, y). Under coherent laser illumination and orderly flow, speckle contrast (k) is approximately related to blood flow as[7]:

$$\mathrm{BF} \propto \frac{1}{k^2} \quad (4)$$

These BF images were resampled to a 30 second time resolution, and co-registered to the IOS images.

Generation of tumor masks

The tumor extent was manually identified using FL images. Corresponding BV images were then used to identify an in-focus FoV for each tumor. These FoVs were used to generate unique masks for each tumor xenograft (**Supplementary Fig. 4**) which were used for subsequent analyses.

Temporal and spatial filtering of multicontrast optical signals

We applied a mean temporal filter (1 minute step size, 5 minute long kernel) to reduce noise levels for each of the three hemodynamic variables BV, Hb_{sat} and BF. This also ensured that all HemoSYS modules operated on hemodynamic time-series with 1 minute temporal resolution. Moreover, the 5 min filter kernel reduced the number of time points available for analysis. For example, a 60 min imaging session yielded 56 time points at 1 min temporal resolution, while a 30 minute session yielded 26 time points at the same temporal resolution.

In addition, all hemodynamic images were subjected to a mean spatial filter with a discrete spatial kernel of 50×50 pixels ensuring that all HemoSYS analyses were performed in 50×50 pixel sub-regions. This helped minimize the computational burden and provided resilience against moderate motion that cab occur in a soft tissue vascular bed such as that of the mammary fat pad. (The propagation analysis module of HemoSYS was the only exception, and used hemodynamic images that underwent mean filtering with a continuous spatial kernel of 50×50 pixels).

References:

1 Schindelin, J. *et al.* Fiji: an open-source platform for biological-image analysis. *Nat Methods* **9**, 676-682, doi:10.1038/nmeth.2019 (2012).

2 Thevenaz, P., Ruttimann, U. E. & Unser, M. A pyramid approach to subpixel registration based on intensity. *IEEE Trans Image Process* **7**, 27-41, doi:10.1109/83.650848 (1998).

3 Hillman, E. M. Optical brain imaging in vivo: techniques and applications from animal to man. *J Biomed Opt* **12**, 051402, doi:10.1117/1.2789693 (2007).

4 Tredan, O., Galmarini, C. M., Patel, K. & Tannock, I. F. Drug resistance and the solid tumor microenvironment. *J Natl Cancer Inst* **99**, 1441-1454, doi:10.1093/jnci/djm135 (2007).

5 Fercher, A. F. Optical coherence tomography - development, principles, applications. *Z Med Phys* **20**, 251-276, doi:10.1016/j.zemedi.2009.11.002 (2010).

6 Rege, A. *et al.* Longitudinal in vivo monitoring of rodent glioma models through thinned skull using laser speckle contrast imaging. *J Biomed Opt* **17**, 126017, doi:10.1117/1.JBO.17.12.126017 (2012).

7 Senarathna, J., Rege, A., Li, N. & Thakor, N. V. Laser Speckle Contrast Imaging: theory, instrumentation and applications. *IEEE Rev Biomed Eng* **6**, 99-110, doi:10.1109/RBME.2013.2243140 (2013).

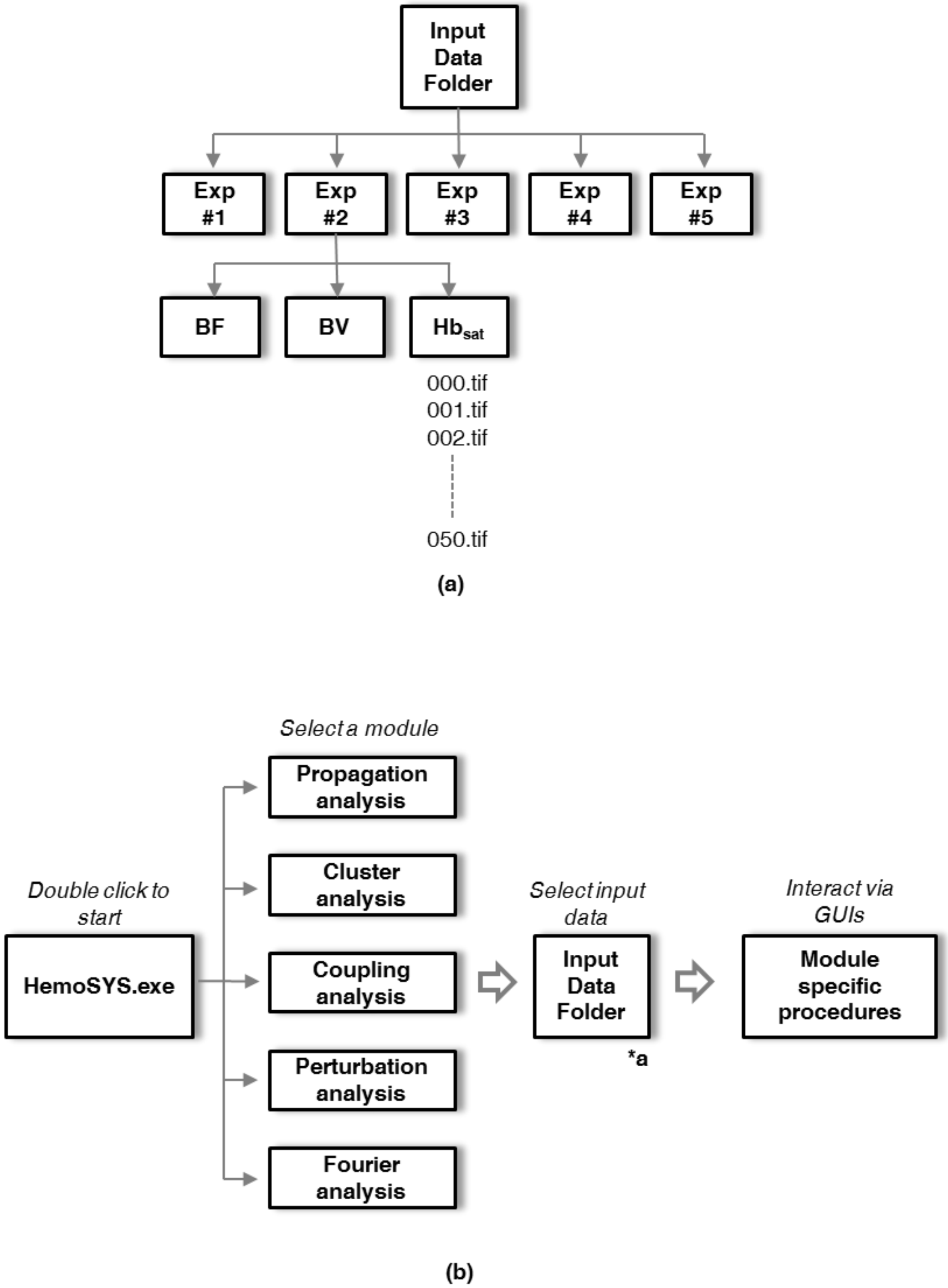

<u>Supplementary Fig. 1: Operation of HemoSYS</u>. (**a**) The recommended input data structure. All image data are stored within a single input data folder. Within this master folder, sub-folders are created to store data for each animal (or experiment). These are named 'Exp #11'… 'Exp

#5’. Users can choose an appropriate prefix to describe their own experiments or trials. Within each experiment or trial folder, additional sub-folders are created for each physiological variable (e.g. BF, BV and Hb_{Sat}). Images corresponding to the time-series of each physiological variable are stored within these sub-folders. Shown here is a list of images under the Hb_{sat} folder using the recommended naming convention for images. (**b**) the HemoSYS command flow structure. User actions are italicized. One begins by double-clicking the HemoSYS.exe icon. The user is then prompted to select one of the five analysis modules, followed by an input data folder (‘* a’ indicates reference to the folder structure in (a)). HemoSYS will automatically recognize the individual trial data and the physiological variables, and prompt the user to proceed in a module-specific manner.

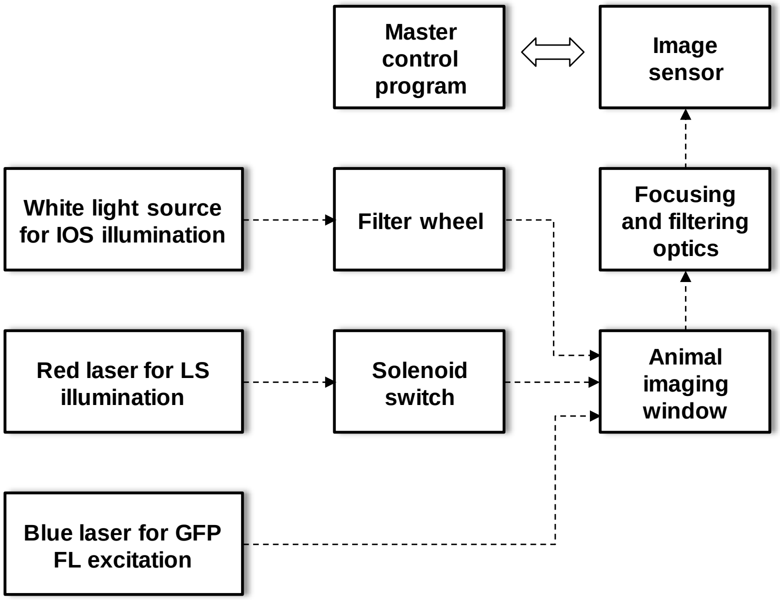

Supplementary Fig 2: Schematic of the multicontrast optical imaging setup. We used three illumination sources: (i) a 473 nm blue laser for the FL excitation source, (ii) a white light source for the IOS illumination, and (iii) a 632.8 nm red He-Ne laser for the LS illumination. The FL

illumination source was manually switched ON/OFF as it was used only once at the beginning of the experiment. The IOS and LS illumination sources were autonomously gated. The IOS illumination source was fitted with a filter wheel containing two bandpass filters (570 nm ± 5 nm and 600 nm ± 5 nm) and a light stop slot. The LS illumination source was fitted with a custom-made light stop switch using a microcontroller actuated solenoid. These were controlled by the master control program running on a personal computer (PC). For clarity, these two controls are not indicated in the schematic. Light from these illumination sources was shone onto the animal imaging window. A lens set was used to focus images on a CCD image sensor. Additionally, a 496 nm longpass filter prevented blue excitation light from entering the imaging light path. The master control program also controlled image acquisition and storage. Dashed arrows indicate light paths, and the hollow bi-directional arrows indicate digital control and data transfer.

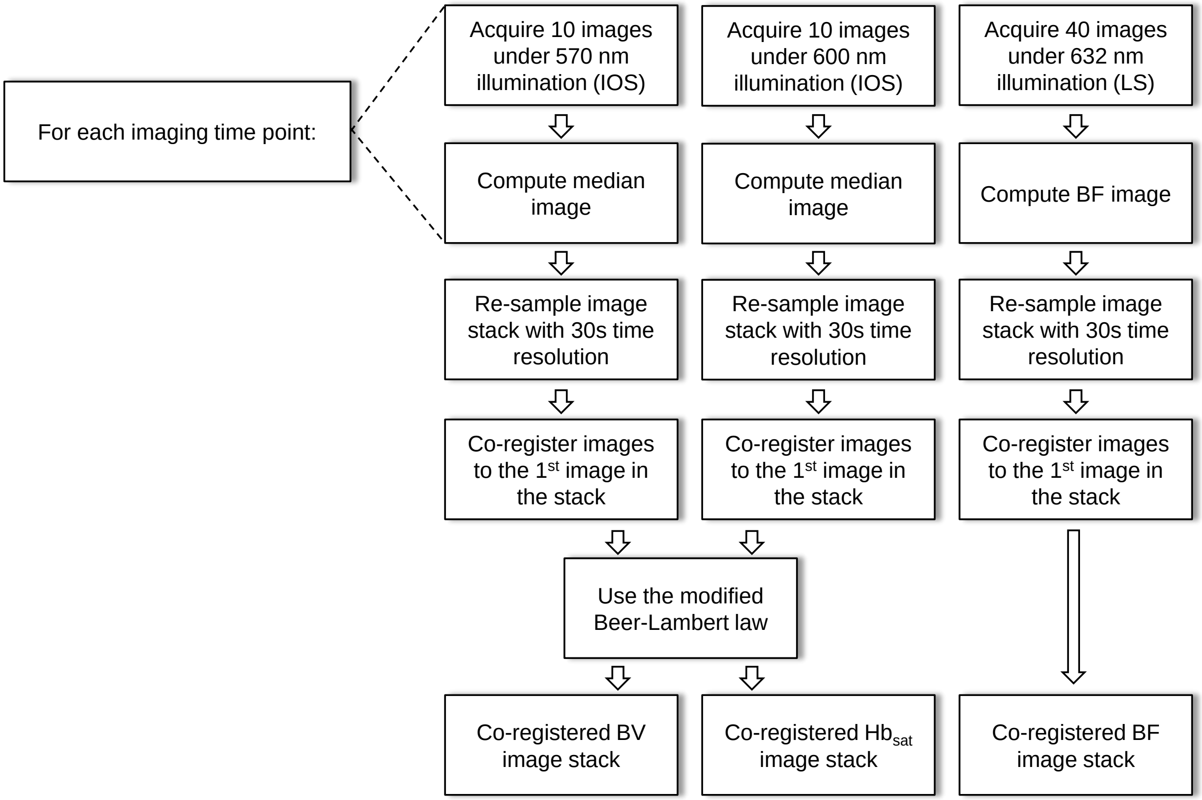

<u>Supplementary Fig. 3: Schematic of the multicontrast pre-processing pipeline</u>.

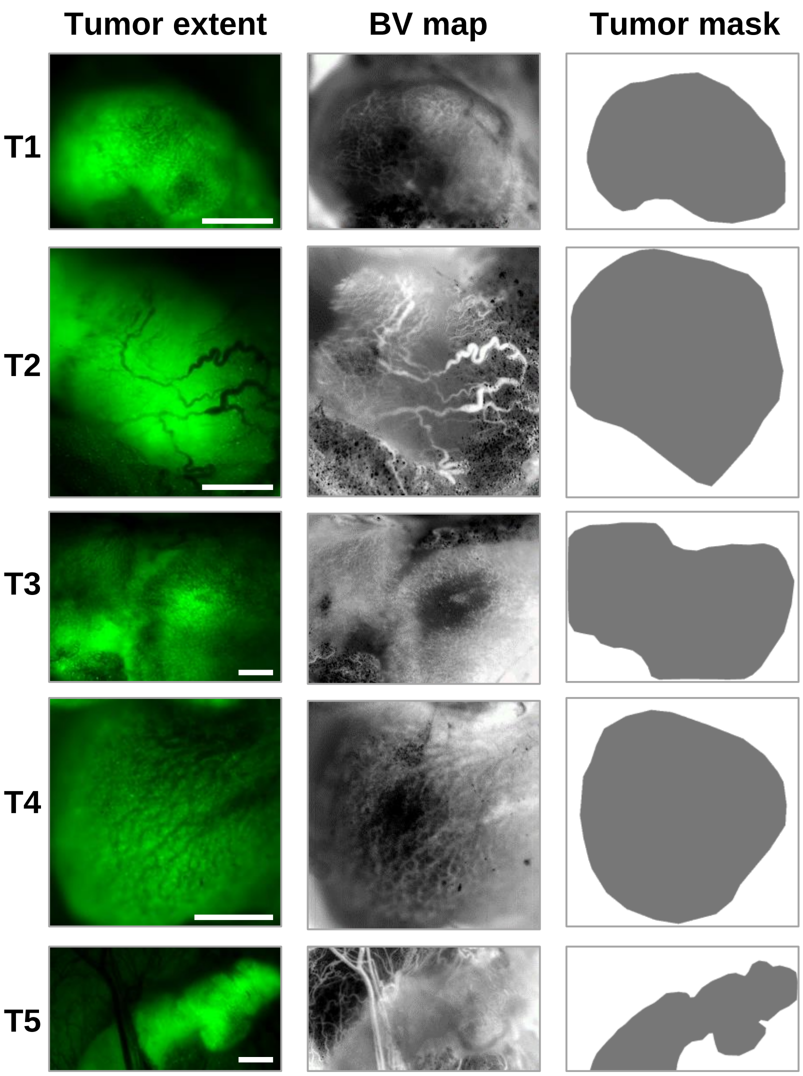

<u>Supplementary Fig. 4: Generation of tumor masks</u>. For each tumor, the tumor extent was manually identified using FL images. The corresponding BV images were then used to identify FoVs that were sufficiently in focus during *in vivo* imaging. Using both the FL image and the BV image, a tumor mask identifying the in-focus tumor extent was manually created for each tumor. Scale bar = 1mm.

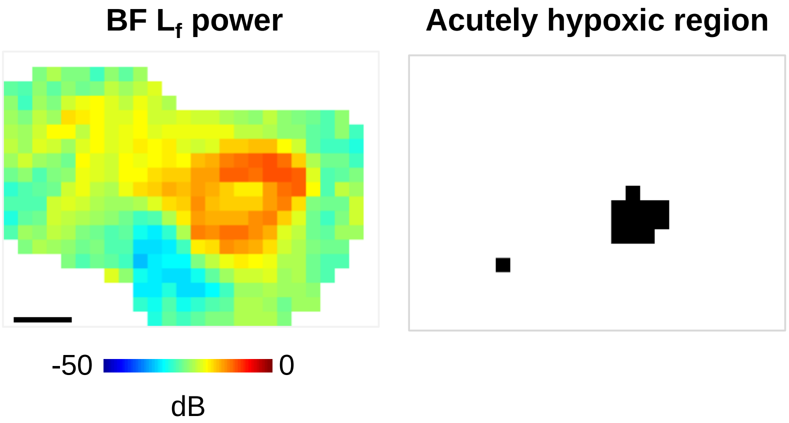

Supplementary Fig. 5: Comparison of the spatial distribution of BF power in the L_f band and the regions that showed acute hypoxia.

	Measurement Techniques	TME Variable	Data Acquisition		Analysis			Notes
			Spatial	Temporal	Spatial	Temporal	Fourier	
Kimura et al 1996[1]	Oxygen microelectrodes, FL	Tissue PO2, BF	Continuous	Continuous, 60 minutes	Discrete locations	Display of continuous waveforms	N/A	Cross variable correlations
Braun et al 1999[2]	Oxygen microelectrodes, LDF	Tissue PO2, BF	Discrete	Continuous, 80 minutes	Discrete locations	Display of continuous waveforms	Yes	Features of frequency spectra, time dependence of these features, and cross variable correlations
Sorg et al 2005[3]	IOS	Hb_{sat}	Continuous	Discrete, days 3,4, and 5	N/A	N/A	N/A	Gas challenges and qualitative analyses
Lanzen et al 2006[4]	Oxygen microelectrodes, FL	Tissue PO2, BF	Discrete PO2, Continuous BF	Continuous, 60 minutes	Discrete locations	Display of continuous waveforms	N/A	Cross variable correlations and plot of tissue PO2 against distance from highest BF vessel
Sorg et al 2008[5]	IOS	Hb_{sat}	Continuous	Continuous, 70 minutes	Discrete locations	Display continuous waveforms	Yes	
Skala et al 2009[6]	IOS, OCT	H_{bsat}, BV, BF, direction of BF and vessel shear stress	Continuous	Discrete, 6 hr sampling points until 24 hr	Discrete locations	Display discrete time point waveforms	N/A	Cross variable scatter plots and correlations
Palmer et al 2010[7]	FL, IOS	Tumor extent, H_{bsat}, Tissue PO2	Continuous	Continuous, 70 minutes	Continuous	Continuous	N/A	Cluster analysis and cross variable correlations
Skala et al 2010[8]	FL, IOS, OCT	H_{bsat}, BV, BF and tissue metabolism	Continuous	Discrete, 6 hr sapling points until 36 hr	Discrete locations	Display discrete time point waveforms	N/A	Plot of tissue metabolism against distance to nearest vessel and cross variable correlations

Supplementary Table 1: Summary of optical imaging-based reports on tumor hemodynamics. For each report, the imaging techniques used, the hemodynamic variables imaged, the spatial and temporal resolutions of image acquisition, the spatial and temporal and frequency paradigms of image analysis, and additional notes are presented. For clarity, reports on static or conventional analyses of tumor angiogenesis (e.g. microvessel density, tortuosity) are not included. FL: Fluorescence imaging, LDF: Laser Doppler flowmetry, IOS: Intrinsic optical signal imaging, OCT: Optical coherence tomography. The blue colored column indicates all instances

where analyses were restricted to selected discrete spatial locations of the TME. The green colored raw shows a single instance where analysis was performed on data that was continuous in the spatial and temporal domains. However, this analysis was limited to oxygenation dynamics.

Supplementary Table References

1 Kimura, H. *et al.* Fluctuations in red cell flux in tumor microvessels can lead to transient hypoxia and reoxygenation in tumor parenchyma. *Cancer Res* **56**, 5522-5528 (1996).

2 Braun, R. D., Lanzen, J. L. & Dewhirst, M. W. Fourier analysis of fluctuations of oxygen tension and blood flow in R3230Ac tumors and muscle in rats. *Am J Physiol* **277**, H551-568, doi:10.1152/ajpheart.1999.277.2.H551 (1999).

3 Sorg, B. S., Moeller, B. J., Donovan, O., Cao, Y. & Dewhirst, M. W. Hyperspectral imaging of hemoglobin saturation in tumor microvasculature and tumor hypoxia development. *J Biomed Opt* **10**, 44004, doi:10.1117/1.2003369 (2005).

4 Lanzen, J. *et al.* Direct demonstration of instabilities in oxygen concentrations within the extravascular compartment of an experimental tumor. *Cancer Res* **66**, 2219-2223, doi:10.1158/0008-5472.CAN-03-2958 (2006).

5 Sorg, B. S., Hardee, M. E., Agarwal, N., Moeller, B. J. & Dewhirst, M. W. Spectral imaging facilitates visualization and measurements of unstable and abnormal microvascular oxygen transport in tumors. *J Biomed Opt* **13**, 014026, doi:10.1117/1.2837439 (2008).

6 Skala, M. C., Fontanella, A., Hendargo, H., Dewhirst, M. W. & Izatt, J. A. Combined hyperspectral and spectral domain optical coherence tomography microscope for noninvasive hemodynamic imaging. *Opt Lett* **34**, 289-291 (2009).

7 Palmer, G. M. *et al.* Optical imaging of tumor hypoxia dynamics. *J Biomed Opt* **15**, 066021, doi:10.1117/1.3523363 (2010).

8 Skala, M. C., Fontanella, A., Lan, L., Izatt, J. A. & Dewhirst, M. W. Longitudinal optical imaging of tumor metabolism and hemodynamics. *J Biomed Opt* **15**, 011112, doi:10.1117/1.3285584 (2010).
