# Supplementary material for: HemoSYS: A Toolkit for Image-based Systems Biology of Tumor Hemodynamics
Source: Sci Rep. 2020 Feb 11;10:2372. doi: 10.1038/s41598-020-58918-3 (PMC7012876; doi:10.1038/s41598-020-58918-3)
Supplement: Supplementary file 2 — Supplementary Information:HemoSYS User Guide [file 41598_2020_58918_MOESM2_ESM.pdf]

HemoSYS: A Toolkit for Image-based Systems Biology of Tumor Hemodynamics

Janaka Senarathna[1], Ayush Prasad[2], Akanksha Bhargava[1], Stacy Gil[1], Nitish V. Thakor[3] and Arvind P. Pathak[1, 3, 4]*

[1]Russell H. Morgan Department of Radiology and Radiological Science, [2]Department of Biophysics, and [3]Department of Biomedical Engineering, [4]Sidney Kimmel Comprehensive Cancer Center, Johns Hopkins University School of Medicine, Baltimore MD 21205

HemoSYS User Manual 1.0

Revision date: 2019-12-18

Installation

1. Download the ‘MyAppInstaller_web.exe’ from http://www.pathaklab.org/HemoSYS/
2. Double click on the .exe icon to start the installation, and follow the prompts for completion.
3. For easy access, search ‘HemoSYS’ in the ‘Start Menu’, right click, and select ‘Pin to taskbar’ or ‘Pin to start’.

Input data structure

HemoSYS accepts sequences of multi-variable image-based datasets. Please organize your input data according to the hierarchy:

Input data folder

↓

A folder for each experiment or trial (e.g. mouse #1, mouse #2 etc.…)

↓

A folder for each physiological variable

↓

Images indicating the spatial distribution of the physiological variable numbered according to their time sequence (i.e. 001, 002, 003 …. 050.tif)

Fig. 1 shows an example input data structure. Here, a cohort of 5 mice (M1…, M5) have been imaged. For each mouse, the physiological variables blood flow (BF), hemoglobin saturation (HbSat) and blood volume (or total hemoglobin, HbT) were concurrently acquired/computed for 56 time points.

Fig. 1a shows the appropriate folder structure. All image sequences are stored within an overall folder called ‘input data’. Within this folder, sub-folders are made to store data for each animal (or experiment). Here, these are named ‘M1’… ‘M5’. Users can choose an appropriate prefix to describe their experiments or trials. Within each such trial or experiment folder, sub-folders are made for each physiological variable (BF, HbSat and HbT). Images corresponding to the time series of each physiological variable are stored inside them. **Fig. 1b** illustrates the image naming convention using HbSat images from M1.

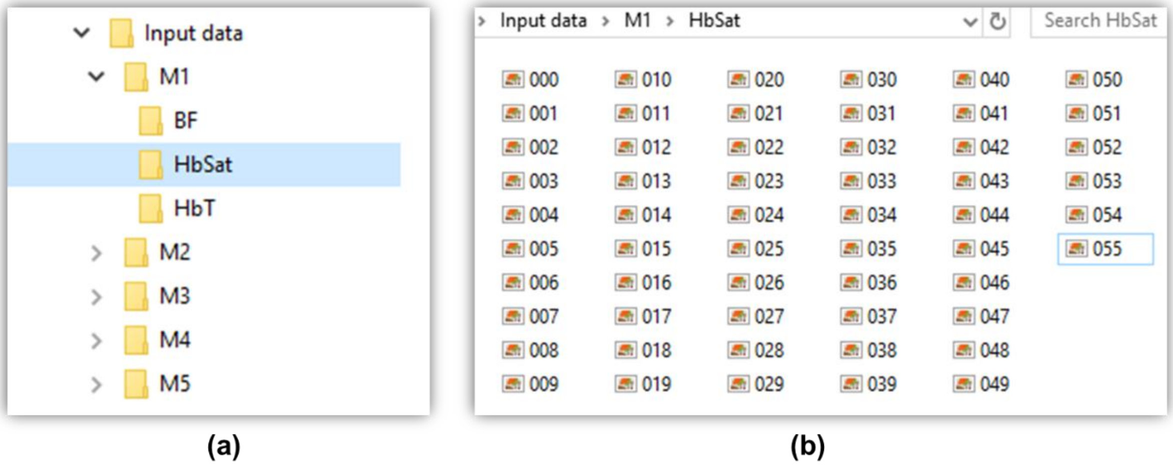

Figure 1: Recommended input data structure. (a) The folder structure, and (b) the suggested image naming convention.

If additional one-time images such as a fluorescence image of tumor extent etc. for the same experiment or trial were also acquired, these can be separately input to HemoSYS on a case by case basis during the runtime.

Starting HemoSYS

1. Double click on the HemoSYS icon to start.
2. A dialog box (**Fig. 2**) will appear.
3. Select the appropriate module (among Propagation, Coupling, Cluster, Perturbation or Fourier), and click ‘OK’ to proceed. Clicking ‘Cancel’ will exit HemoSYS.

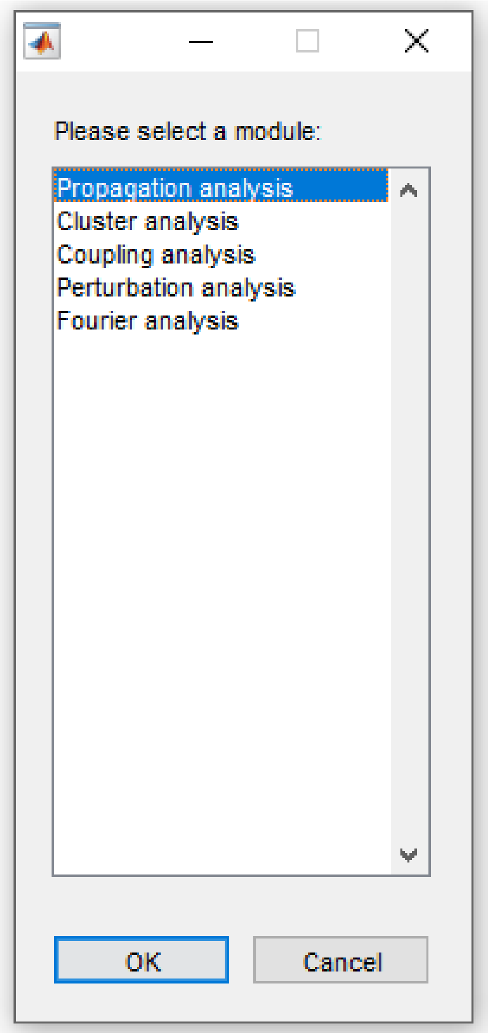

Figure 2: Prompt to select which module to use.

Propagation Analysis Module

1. A dialog box will appear prompting the user to select the input data folder.
2. Please locate and select the appropriate input data folder (as described previously) and click 'Select Folder'. HemoSYS will automatically recognize the data structure.
3. A dialog box will appear prompting the user to select the experiment or trial on which to run the propagation analysis (**Fig. 3**).

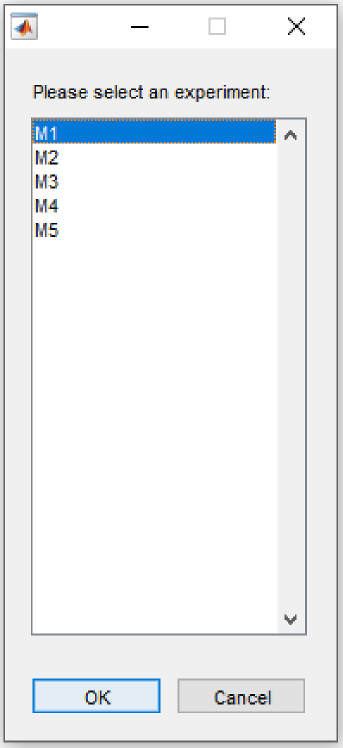

Figure 3: Prompt to select the experiment or trial for processing.

4. Please select the appropriate experiment and click the 'OK' button. For illustration, we selected M3.
5. A dialog box will appear prompting the user to select any 'one-time' (i.e. non-time-series) images that represent the experiment or trial (**Fig. 4**). For example, the cohort of animals used here were inoculated with tumor cells that were tagged with a green fluorescence protein (GFP) indicator. Here, the GFP fluorescence (FL) image showing the tumor extent can be loaded into HemoSYS as an example 'one-time' image for the experiment or trial.

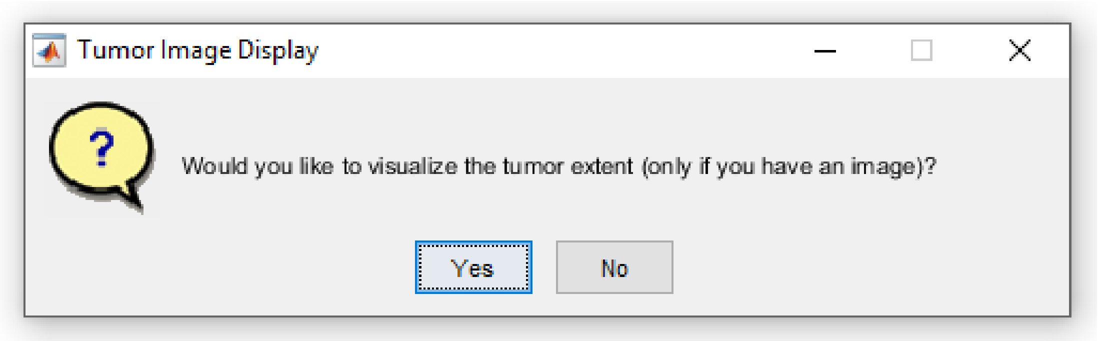

Figure 4: Prompt to select a one-time (e.g. tumor extent) image for the experiment or trial.

6. If you click 'Yes', a dialog box similar to that in Step 1 will appear to enable selecting the image. The user can also clock 'No' and skip this step.
7. A dialog box prompting the user to select a physiological variable will appear (**Fig. 5**). Note: HemoSYS automatically detects the list of physiological variables from the input data structure.

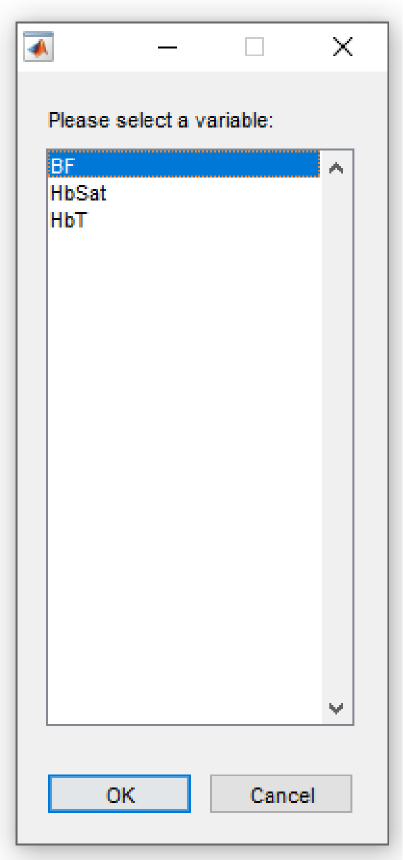

Figure 5: Prompt to select a physiological variable for analysis.

8. Select the appropriate physiological variable and click ‘OK’. For demonstration purposes, we select ‘HbSat’.
9. A dialog box will appear prompting the user to provide the temporal resolution of the image sequence as well as the value range to display images (**Fig. 6**). Please click ‘OK’ after typing in the appropriate values. Here we inserted 1 min as the temporal resolution, and 0 – 80 as the display range.

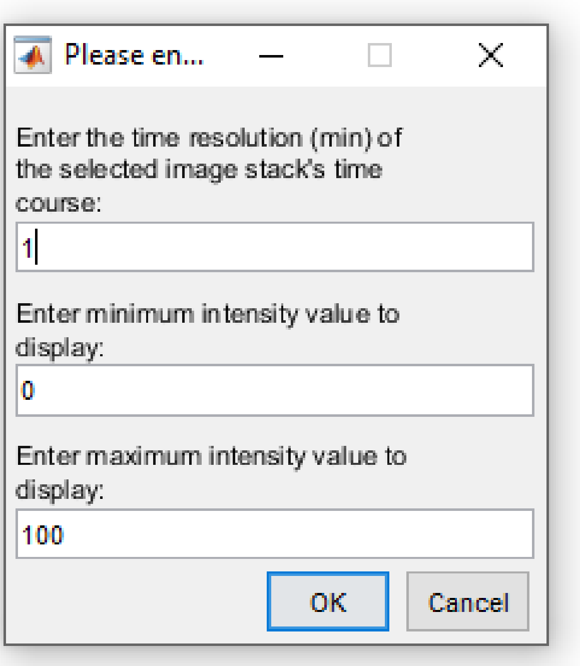

Figure 6: Prompt to input temporal resolution of input images and the display range.

10. The user will be prompted to select an output folder to save various outputs that will be generated by the module (**Fig. 7**). Please click ‘OK’ to proceed. By default, HemoSYS will create a folder named ‘Outputs’ in the corresponding experiment or trial folder. However, the user can navigate and select a folder of their choice as the output folder.

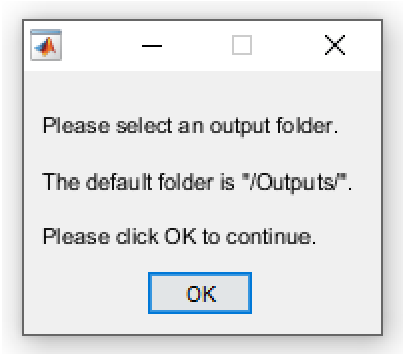

Figure 7: Prompt to select an output folder.

11. The first image of the selected physiological variable will be displayed over the specified range of intensities. A ‘jet’ color map is used (e.g. **Fig. 8**).

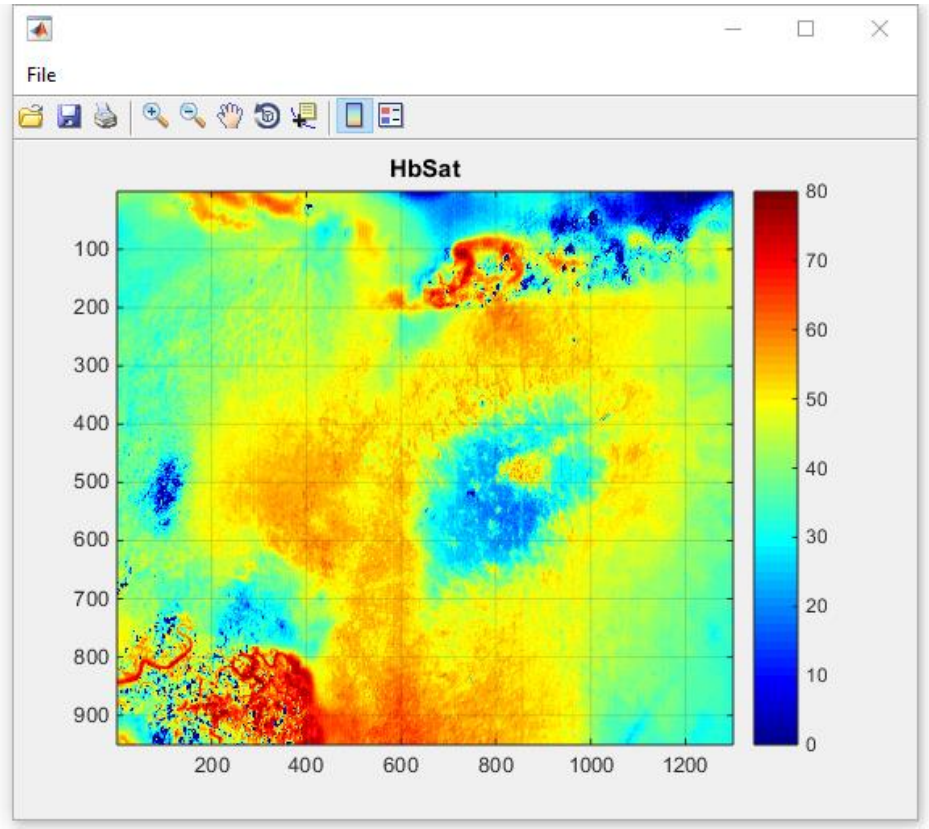

Figure 8: The first image of the selected physiological variable is displayed over the specified range of intensities.

12. A dialog box will then appear prompting the user to select a region of interest (ROI) for subsequent analysis (**Fig. 9**).

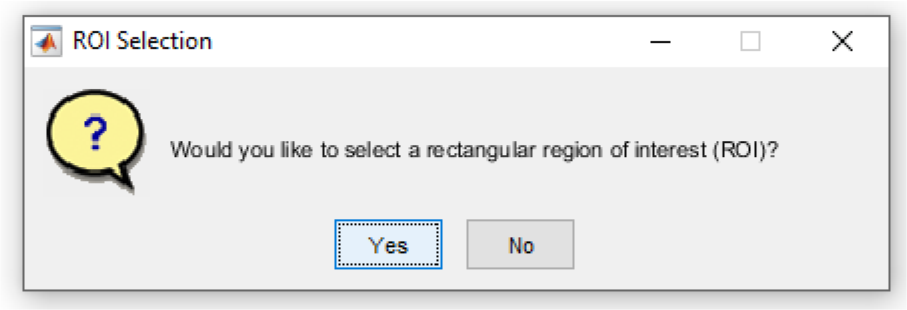

Figure 9: Prompt to select a region of interest (ROI) for further analysis.

13. If ‘Yes’ is selected, please mark the top left and the bottom right corners of the desired ROI. HemoSYS will display the following notification (**Fig. 10**) to ensure proper ROI selection. Please click ‘OK’ to proceed to selecting the ROI.

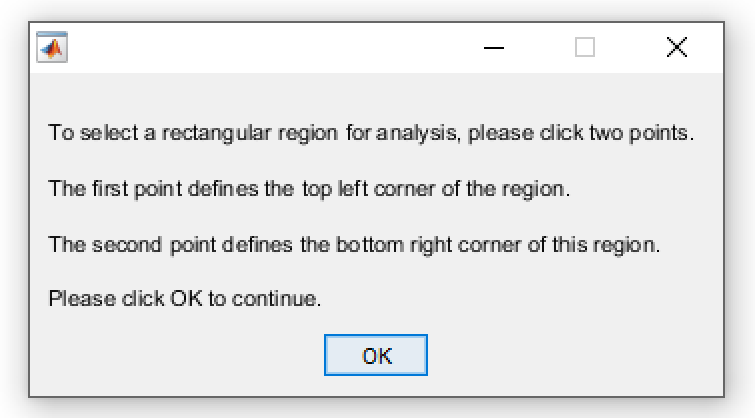

Figure 10: Notification detailing how to select an ROI.

14. Note: If a proper sequence of coordinates are not chosen (i.e. a top left corner and a bottom right corner), HemoSYS will automatically detect this and prompt the user to reselect the ROI.
15. The selected ROI will be indicated by a dashed box within the image (e.g. **Fig. 11**).

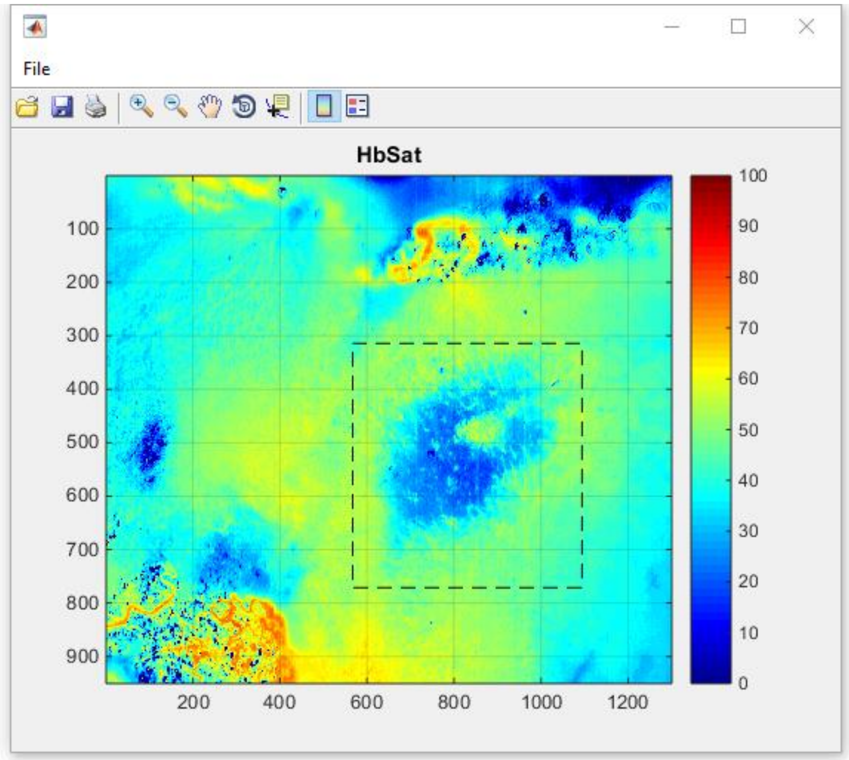

Figure 11: The selected ROI will be displayed with a dashed outline.

16. A dialog box will appear to provide the user the option to either reselect a new ROI or continue with the present ROI (**Fig. 12**).

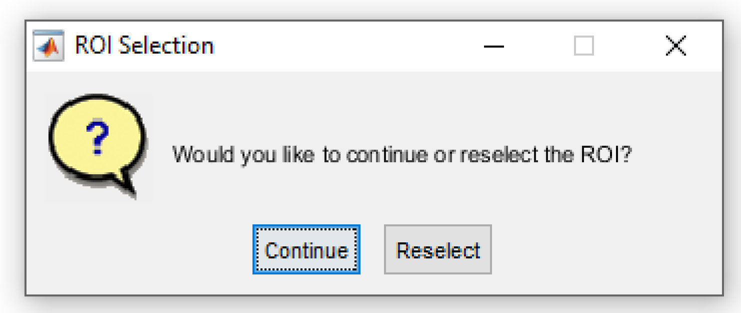

Figure 12: Prompt to either select a new ROI or continue with the present selection.

17. Conversely, one can click ‘No’ in step 12, and the entire image space will be considered for analysis.
18. A dialog box will appear providing the user with the option to visualize time lapse images of the selected ROI (**Fig. 13**).

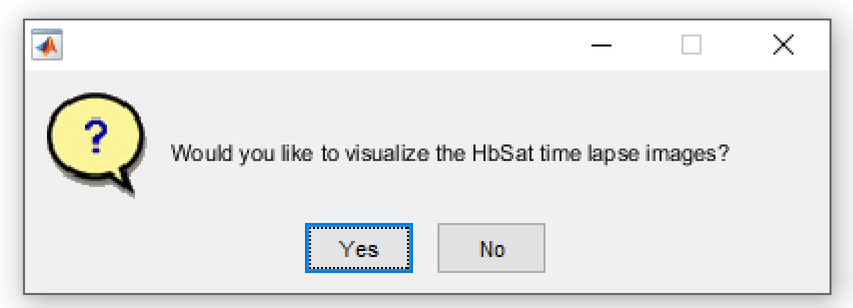

Figure 13: Prompt for visualizing time lapse images.

19. The user can opt to skip this step by clicking 'No'. If 'Yes' is clicked, then a subsequent window will appear requesting values for the gap between subsequent images to be displayed and the range of values to display these images (**Fig. 14**). For illustration we select a 10 min gap and a value range of 0-70.

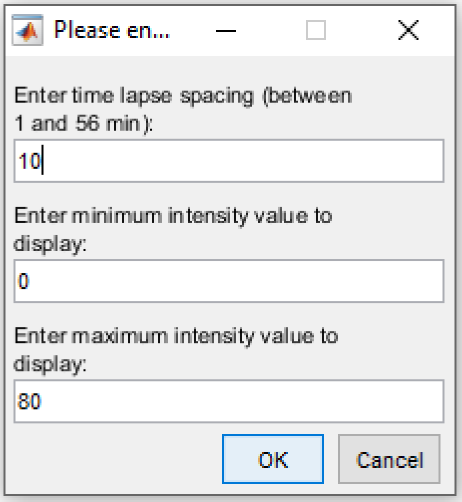

Figure 14: Prompt for entering time gap between consecutive time lapse images and the value range to be displayed.

20. **Fig. 15** shows an example time lapse image dataset created by HemoSYS.

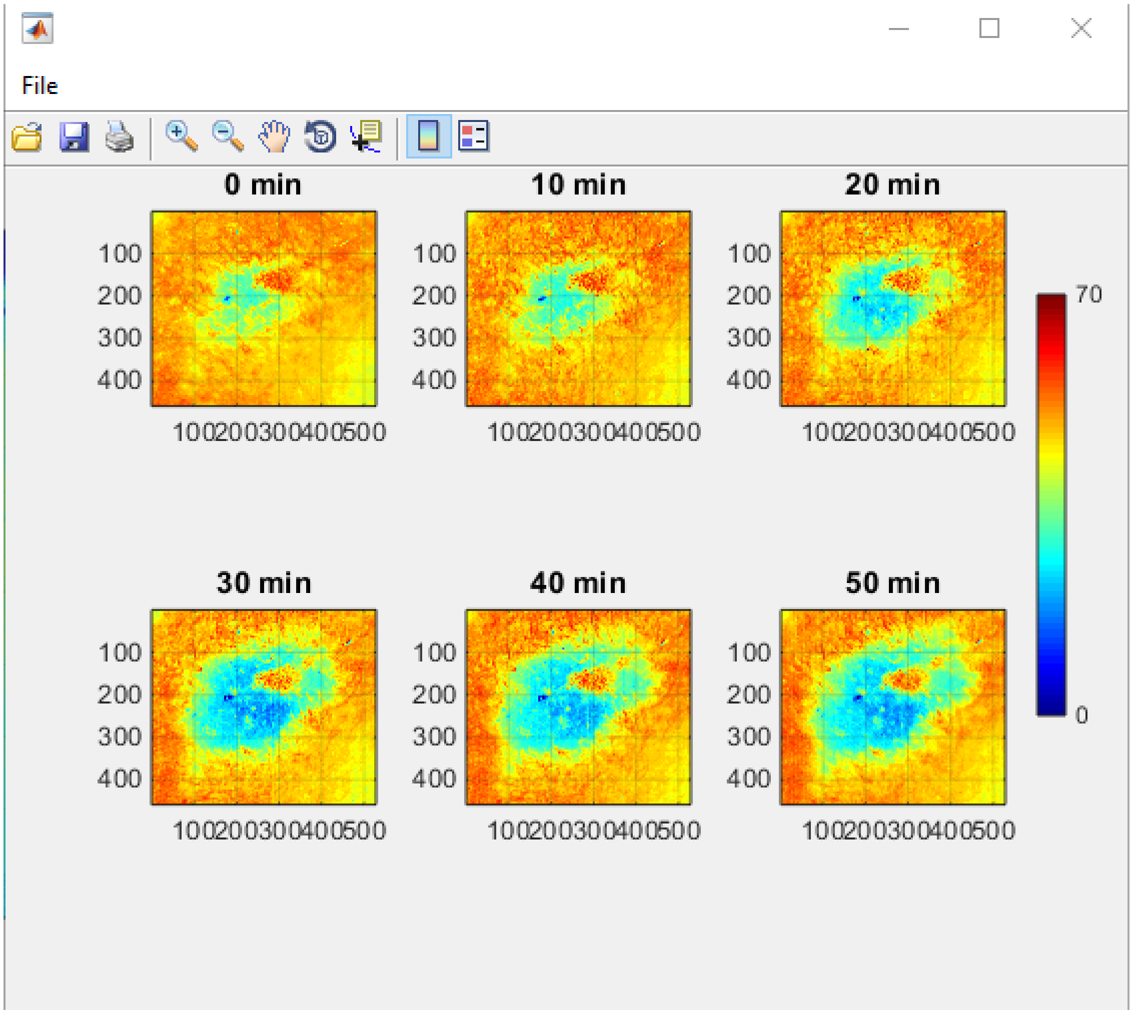

Figure 15: Example time lapse visualization.

21. Furthermore, these images will be automatically saved in the ‘Output’ folder selected previously.
22. The user will be also be provided with an option to revisualize time lapse images with a different set of parameters (**Fig. 16**). Once satisfied, the user can click ‘Continue’ to proceed.

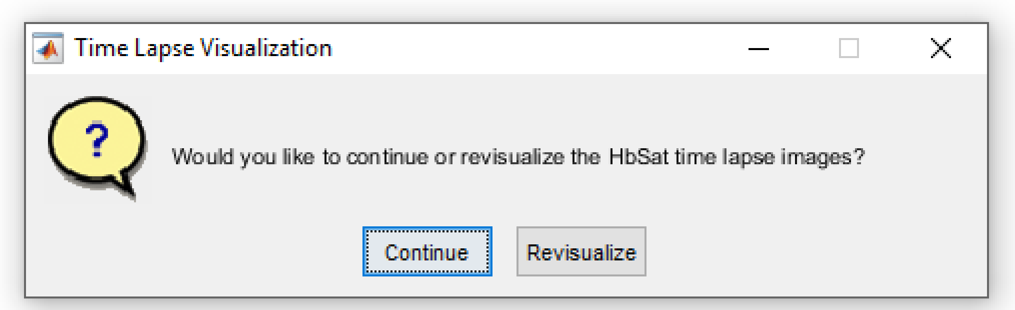

Figure 16: Prompt for re-visualizing time lapse data.

23. The proceeding steps in the Propagation Analysis Module will aim to characterize propagation dynamics. Therefore, the user will be prompted to enter several specifications associated with visualizing propagation dynamics (**Fig. 17**). These include the smoothing kernel size (radius) needed for smoothing the data prior to the propagation analysis, the threshold value to be used for analysis (e.g. for HbSat, what oxygen saturation level to be used for assuming a hypoxic condition), and the time step size to plot contours where the threshold is exceeded. For illustration, we entered a kernel size of 50 pixels, a threshold of 30% and a time step size of 1 min for displaying the contour plots.

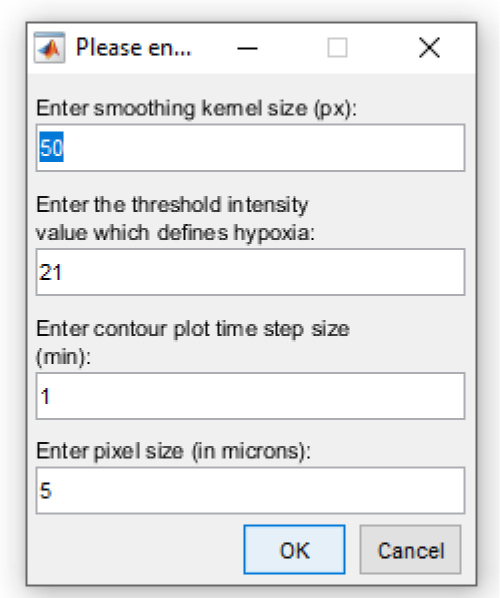

Figure 17: Prompt to enter specifics for visualizing propagation dynamics.

24. For clarity contour plots will be shown in both a jet color map and a fire color map with white background (**Fig. 18**). Additionally, the contour plot will be automatically saved in the 'Output' folder.

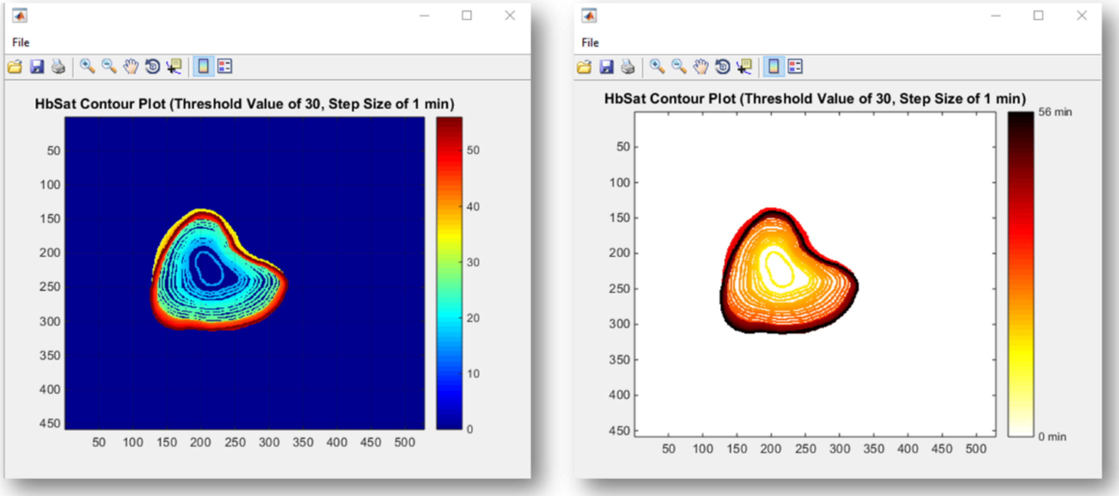

(a) (b)

Figure 18: Contour plots in (a) a jet colormap and (b) a fire colormap.

25. A dialog box will appear providing the user with the option to redo the contour plot generation with different specifications (**Fig. 19**). Please click ‘Continue’ after contour plots have been generated to satisfaction.

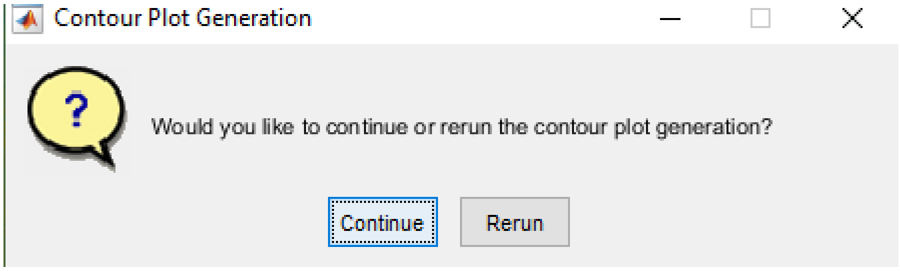

Figure 19: Prompt for rerunning the contour plot generation.

26. A dialog box will then appear prompting the user to select two locations for plotting propagation speeds (**Fig. 20**).

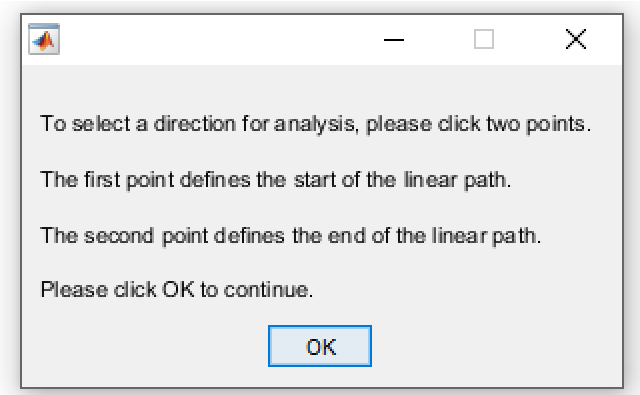

Figure 20: Prompt to select path for propagation speed analysis.

27. Please click ‘OK’, wait until the cross hairs appear and select start and end points of a linear path to visualize propagation speeds. **Fig. 21** shows an example.

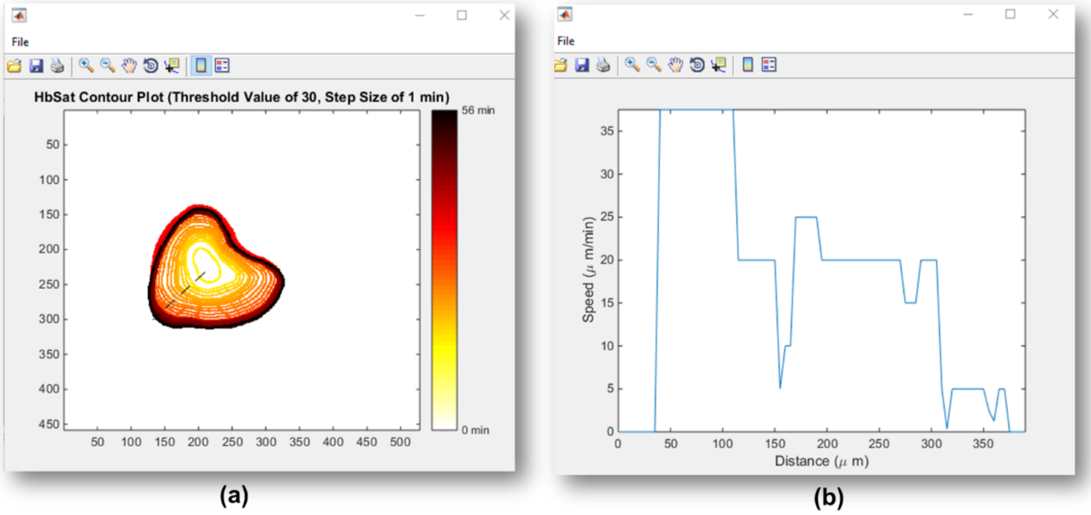

(a) (b)

Figure 21: Propagation dynamics. (a) Contour plot with the selected path marked as a dashed line, and (b) the corresponding propagation speed vs distance plot.

28. The user will be prompted via another dialog box to select a new path and generate a new propagation plot (**Fig. 22**).

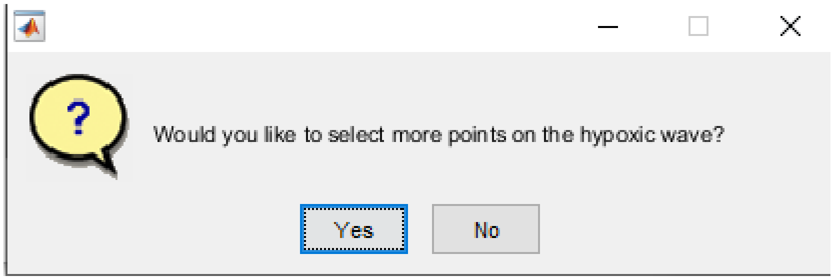

Figure 22: Prompt for rerunning the path selection and propagation speed graph generation.

29. Once, the above step is done, the user will be prompted to select two variables for generating a scatter plot to compare normal (i.e. normoxic, in this example) vs. abnormal (i.e. hypoxic in this example) regions (**Fig. 23**).

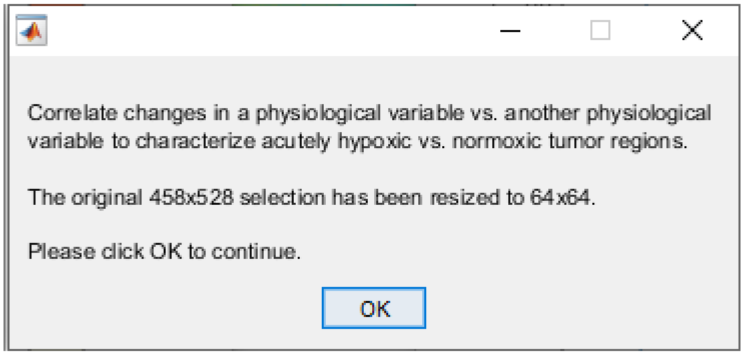

Figure 23: Prompt for selecting two variables.

30. Once, 'OK' is clicked, the user will be prompted further to select each additional variable. HemoSYS will then generate a scatter plot comparing normal vs abnormal regions (e.g**. Fi. 24**). An accompanying dialog box (**Fig. 25**) will provide the appropriate legend.

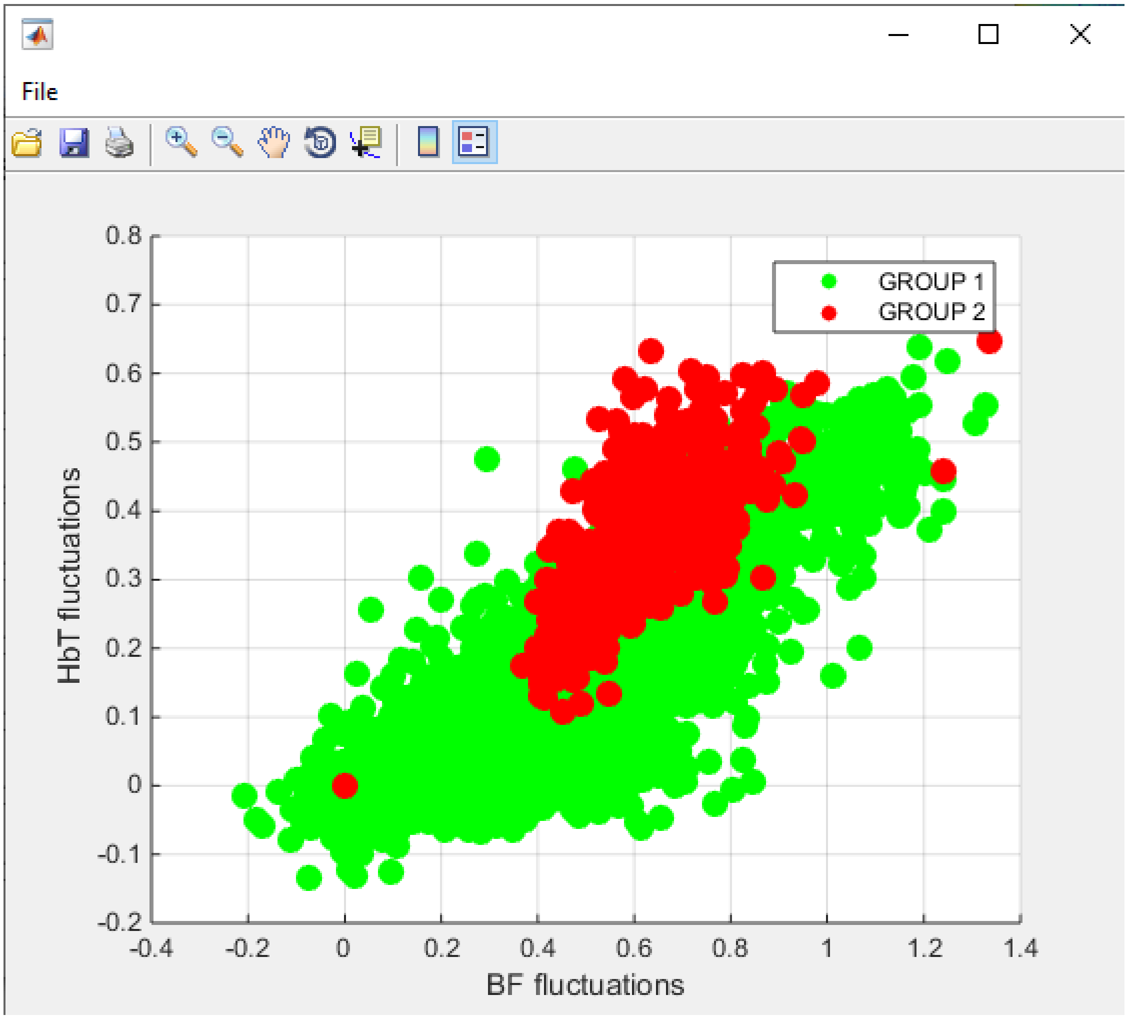

Figure 24: Example scatter plot. Each axis is the standard deviation/mean over the entire time period.

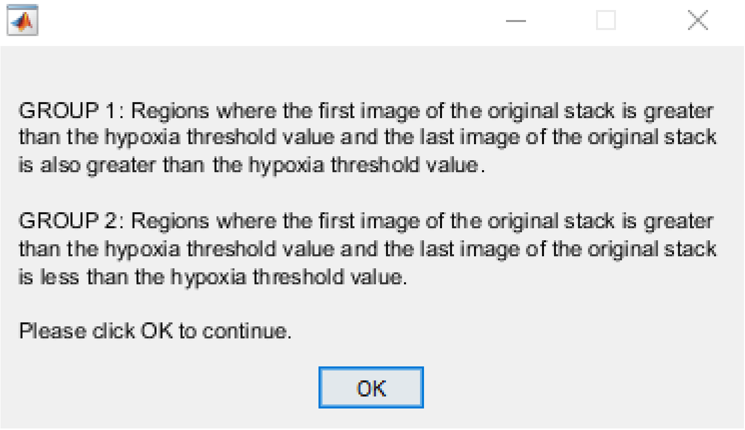

Figure 25: Legend for the scatter plot.

31. At this point, the propagation analysis is complete. The user will be prompted to either chose to either rerun the propagation analysis module or end the analysis (**Fig. 26**).

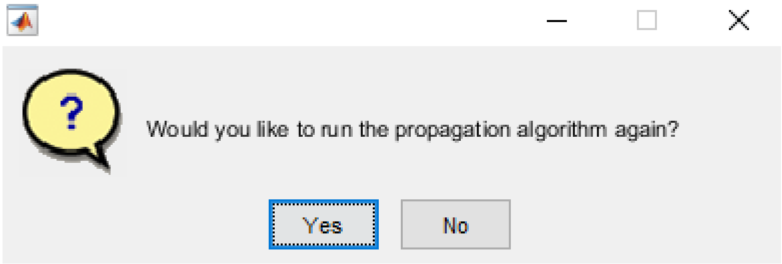

Figure 26: Prompt to rerun the propagation analysis.

32. If ‘No’ was chosen, then the user can quit HemoSYS. Double click again on the HemoSYS icon to run a different analysis module.

Rest of the Modules

The rest of the analysis modules are operated in the same manner as the propagation analysis module.
